# Optimizing age-structured sampling for estimating the seroconversion rate in malaria seroepidemiology: a simulation study

**DOI:** 10.1038/s41598-026-55736-x

**Published:** 2026-05-30

**Authors:** Yura K. Ko, Shilei Li, Tom Britton, Wataru Kagaya, Akira Kaneko

**Affiliations:** 1 Department of Microbiology, Tumor and Cell Biology (MTC), Karolinska Institutet, Stockholm, Sweden; 2 Department of Vector Ecology and Environment, Institute of Tropical Medicine (NEKKEN), Nagasaki University, Nagasaki, Japan; 3 Department of Mathematics, Stockholm University, Stockholm, Sweden; 4 Department of Ecoepidemiology, Institute of Tropical Medicine (NEKKEN), Nagasaki University, Nagasaki, Japan; 5 Osaka International Research Center for Infectious Diseases, Graduate School of Medicine, Osaka Metropolitan University, Osaka, Japan

**Keywords:** Malaria, Seroprevalence, Reverse catalytic model, Seroconversion rate, Allocation, Precision, Diseases, Health care, Medical research

## Abstract

Seroprevalence and the seroconversion rate (SCR) are key indicators of malaria burden, particularly in low-transmission settings. Optimizing age-based sampling may improve the efficiency and precision of SCR estimation, but this has not been systematically explored in malaria seroepidemiology. We employed Monte Carlo simulations to identify optimal age-based sampling strategies under varying assumptions by modifying (1) SCR, (2) seroreversion rate (SRR), and (3) whether SRR was known or unknown. Using the reverse catalytic model and its extension, we considered two transmission scenarios: a stable SCR and a reduction in SCR at a defined change point. Realistic ranges for SCR and SRR were selected based on previous reports, assuming serological responses to *Plasmodium falciparum* MSP1. In stable settings, sampling older individuals improved precision in low-transmission settings, whereas younger age groups were more informative in high-transmission settings when SRR was known. When SRR was unknown, sampling more younger individuals and maintaining a balanced age distribution yielded higher precision. Under changing transmission, sampling individuals born after the change point improved estimation of post-change SCR, while balanced sampling performed best when SRR was unknown. To illustrate the practical implications of these findings, we applied the framework to published malaria serological data from Sri Lanka. The original study employed an age distribution heavily skewed toward adults, resulting in reduced precision for the post-change SCR and limited statistical power to detect a change in transmission. Our simulations demonstrate that alternative age-based sampling strategies could substantially improve both precision and power under comparable conditions. These findings highlight the importance of careful sampling design in enhancing study efficiency, particularly in settings with limited personnel or financial resources, and emphasize that age-structured sampling strategies should be thoughtfully considered to ensure adequate precision when estimating SCR from a single cross-sectional malaria serological survey.

**Supplementary Information:**

The online version contains supplementary material available at 10.1038/s41598-026-55736-x.

## Introduction

Despite continuous efforts in prevention and treatment over decades, malaria remains a global health challenge as progress in reducing cases and deaths has stalled since 2015^1^. While several countries have been certified malaria-free by the World Health Organization (WHO) in recent years, including Azerbaijan, Tajikistan, and Belize in 2023 and Cabo Verde and Egypt in 2024, the current malaria situation remains complex. In malaria-endemic countries, there is a highly heterogeneous transmission intensity, while some areas experience very low malaria transmission^2^.

Indicators such as *Plasmodium* parasite prevalence and the entomological inoculation rate are commonly used to estimate malaria risk. However, these indicators have low precision and require large sample sizes for accurate estimation in low-transmission settings or pre-elimination settings^3^. Meanwhile, serological markers of *Plasmodium* species-specific antibodies capture past infections, making the seroconversion rate (SCR) a useful alternative for estimating transmission intensity^4^.

The SCR is commonly estimated using the reverse catalytic model (RCM). This model assumes that SCR remains uniform for all individuals in a population across time periods. In addition, the model incorporates a seroreversion rate (SRR), which reflects the waning of antibody responses over time in the absence of exposure, and is influenced by host-related factors affecting antibody dynamics^5^. In recent years, there have been further approaches to estimating transmission intensity using serological markers on the basis of RCM. Yman et al. (2016) used antibody acquisition models, which leverage continuous antibody levels rather than dichotomizing individuals into seropositive or seronegative categories^6^. van den Hoogen et al. (2015)^7^ and Varela et al. (2020)^8^ proposed models assuming multiple change points in SCR, while Kyomuhangi et al. (2021)^9^ developed a method combining RCM and antibody acquisition models. Nevertheless, recent studies continue to use either the simple constant-SCR model or a model with a single change-point assumption^10,11^ due to their relative simplicity.

Sample size calculators for estimating SCR using RCM under stable transmission intensity were proposed by Sepúlveda and Drakeley in 2015^12^. The method for sample size determination was further extended to detect changes in transmission intensity, estimating the transmission change point and the SCR values before and after that point^13^. Such changes in transmission intensity often reflect the impact of malaria control interventions, such as the introduction or scale-up of vector control measures or mass drug administration^14^. The findings from these studies suggested that large sample sizes are required to estimate SCR in low transmission settings with an unknown SRR— the most common context in malaria seroepidemiological surveys. One possible approach to reducing the required sample size is the implementation of an efficient age-sampling framework. Blaizot et al. showed that the optimal allocation varied depending on the epidemiological parameter of interest and the target diseases, such as measles, mumps, and rubella^15^. While there is a growing literature on malaria seroprevalence, including approaches to improve estimation precision using quantitative antibody responses, these studies have not explicitly addressed the problem of optimal age allocation in sampling design^16^. To our knowledge, the question of how to efficiently allocate samples across age groups to maximise precision remains underexplored in the context of malaria.

In the present study, we aim to propose an optimal age-based sampling framework to achieve higher precision in SCR estimation for malaria seroepidemiological surveys using realistic scenarios. To support this goal, we developed a Shiny application that enables users to obtain optimal age allocations based on their specified assumptions.

## Methods

### Models

We briefly present the general concept of the RCM and its extension; more in-depth explanation can be found in Drakeley et al.^17^ and Sepúlveda et al.^13^. Assuming a closed population with constant SCR (denoted by $\:\lambda\:$) and SRR (denoted by $\:\rho\:$), we obtain the following ordinary differential equation:

$$\:\frac{dp\left(a\right)}{da}=\lambda\:(1-p(a\left)\right)-\rho\:p\left(a\right)$$

where $\:p\left(a\right)$ is the propotion of seropositive individuals at age *\:a*. The solution to this equation gives the age-specific seroprevalence:

1 $$\:p\left(a\right)=\:\frac{\lambda\:}{\lambda\:+\rho\:}(1-{exp}\left(-\left(\lambda\:+\rho\:\right)a\right)$$

An extension of the RCM assumes a single change point in the SCR while keeping the SRR constant over time (hereafter referred to as RCM-scSCR)^13^. The seroprevalence $\:{p}^{*}\left(a\right)$ is then obtained by:

2 $$\:{p}^{*}\left(a\right)=\left\{\begin{array}{c}\frac{{\lambda\:}_{2}}{{\lambda\:}_{2}+\rho\:}(1-{exp}\left(-\left({\lambda\:}_{2}+\rho\:\right)a\right)),a\le\:\tau\:\\\:\frac{{\lambda\:}_{2}}{{\lambda\:}_{2}+\rho\:}(1-{exp}\left(-\left({\lambda\:}_{2}+\rho\:\right)\tau\:\right))+\frac{{\lambda\:}_{1}}{{\lambda\:}_{1}+\rho\:}(1-{exp}\left(-\left({\lambda\:}_{1}+\rho\:\right)\left(a-\tau\:\right)\right)){exp}\left(-\left({\lambda\:}_{2}+\rho\:\right)\tau\:\right),a>\tau\:\end{array}\right.$$

where, $\:{\lambda\:}_{1}$ and $\:{\lambda\:}_{2}$ are the SCRs before and after the change point, respectively, and $\:\tau\:$ is the time (in years) since the change point. $\:{p}^{*}\left(a\right)$ also depends on the change point time $\:\tau\:$; however, since we do not consider different values of $\:\tau\:$, we suppress this dependence in the notation. The model is commonly employed to identify abrupt historical reductions in disease transmission, typically following the implementation or scale-up of malaria control interventions^14,18^. For example, Kaneko et al. analyzed seroprevalence data from Aneityum Island in Vanuatu using the RCM-scSCR model and demonstrated that the change point in SCR coincided with a decline in transmission following the implementation of an integrated elimination program combining mass drug administration and vector control^14^.

To estimate the SCR and SRR from observed data, we used maximum likelihood estimation (MLE) based on the following log-likelihood function^19^:

$$\:l\left(p\right)=\sum\limits_{i=1}^{n}{y}_{a,\:i}{log}{p}_{a,\:i}+\sum\limits_{i=1}^{n}{(1-y}_{a,\:i}){log}(1-{p}_{a,i})$$

where $\:{y}_{a,\:i}$ is the binary outcome ($\:{y}_{a,\:i}$ = 1 if seropositive and $\:{y}_{a,\:i}$ = 0 if seronegative) for individual *\:i* at age *\:a*, $\:{p}_{a,i}$ corresponds to either $\:p\left(a\right)$ or $\:{p}^{*}\left(a\right)$, as defined in Eq. (1) or (2), respectively. Parameter estimation was performed by MLE with respect to $\:\lambda\:$ and $\:\rho\:$ (or $\:{\lambda\:}_{1},\:{\lambda\:}_{2}$, and $\:\rho\:$ if using $\:{p}^{*}\left(a\right)$) and implemented in Julia using the Optim.jl package. For models with a single parameter, we used the Brent method, whereas for models with two or more parameters, we used the Nelder–Mead algorithm. In practice, when jointly estimating the SCR and SRR, strongly biased sampling strategies, such as including only individuals above a certain age, may lead to inflated SRR estimates. To avoid implausible estimates of SRR, we constrained the parameter space during optimization by restricting $\:\rho\:$ to values between 0 and 0.1, based on previously reported ranges^7,10,20^ by using a box-constrained optimization algorithm. For RCM-scSCR, we assumed the case of a known change point because a previous study suggested that the antibody data taken as a binary outcome might not have sufficient information to estimate the true change point with high precision^13^.

### Scenarios and estimating parameters

**Fig. 1:**
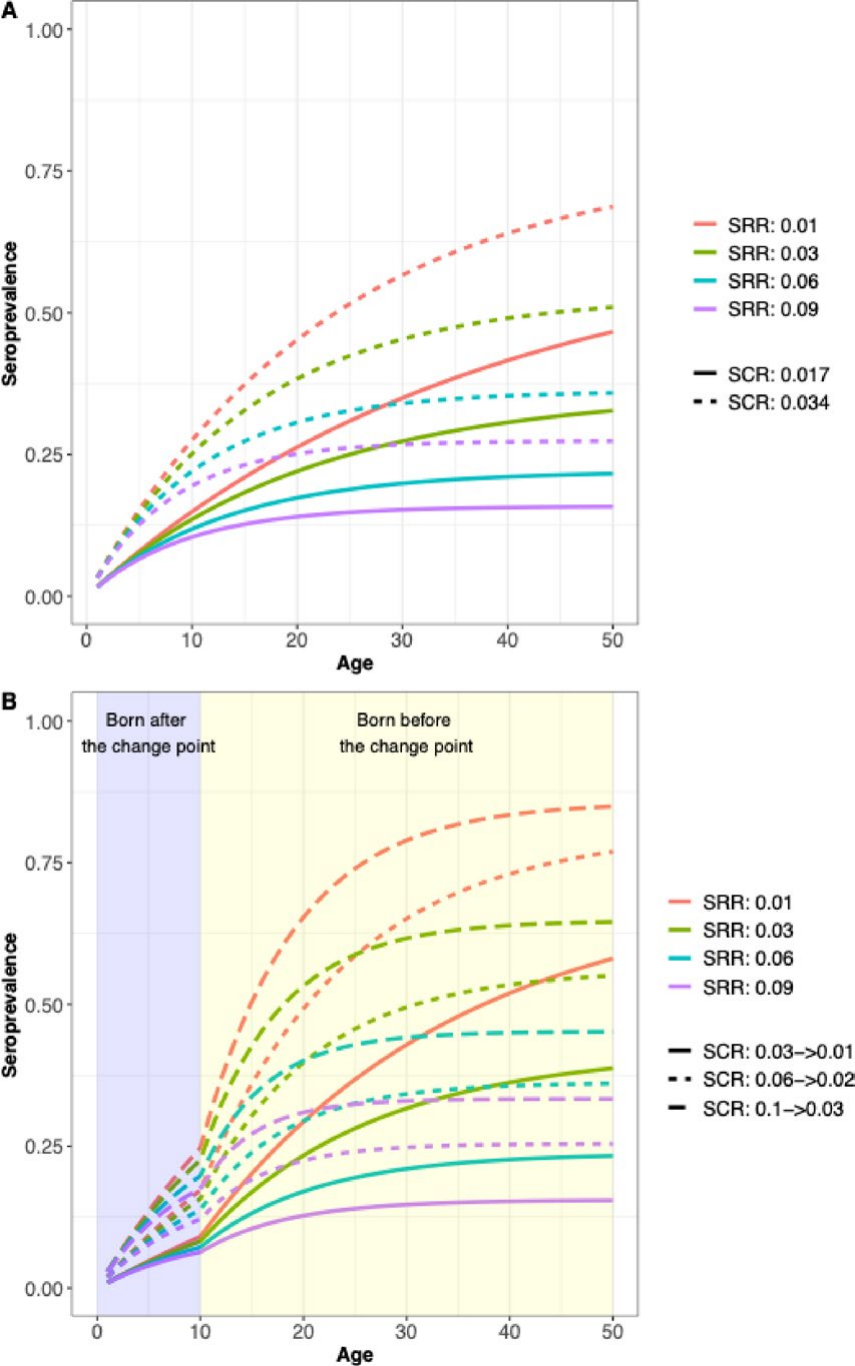
Age-specific seroprevalence curves across different combinations of seroconversion rate (SCR) and seroreversion rate (SRR): **(A)** estimated using the reverse catalytic model (RCM), and **(B)** estimated using the RCM with a single change point in SCR (10 years before the sampling).

We explored two scenarios: (1) assuming a stable SCR (Scenario 1) and (2) assuming a reduction in SCR (Scenario 2). To illustrate how the models link parameters to observed data, we first examined age-specific seroprevalence curves under different combinations of SCR and SRR (Fig. 1). These figures illustrate that age-specific seroprevalence patterns are influenced by both SCR and SRR, such that different parameter combinations can yield similar observed patterns.

Based on this framework, we then evaluated sampling and estimation strategies under each scenario. In each scenario, we varied the SCR(s), SRR, and $\:\tau\:$ (only for scenario 2) to identify the optimal age sampling structures under each set of assumptions. Additionally, we conducted two estimation strategies: estimating only SCR(s) or jointly estimating both SCR(s) and SRR. Table 1 summarizes the models used, underlying assumptions, and parameters estimated for each scenario. Realistic ranges for SCR and SRR were selected based on previous reports from different countries^7,10,20^, assuming the corresponding values pertain to *Plasmodium falciparum* merozoite surface protein 1 (MSP1). Specifically, SCR values of 0.01 and 0.1 were considered to represent low and high transmission settings, respectively. In Scenario 2, pre- and post-change SCR values included combinations such as (λ₁, λ₂) ∈ {(0.03, 0.01), (0.1, 0.03)}, representing transitions from low to very low transmission and from high to low transmission, respectively. SRR values of 0.017 and 0.05 were considered across all simulation settings. For further combinations of sample size N and assumptions of SCR(s), SRR, and $\:\tau\:$, a Shiny application is available (See the Supplementary file for detailed instructions on using the application).

**Table 1:** Summary of the model, assumption, and estimated parameters for each scenario.

Scenario	Model	Assumptions	Estimated parameters
Scenario 1	RCM	$\:\lambda\:=\{0.01,\:0.1\},$ $\:\rho\:=\{0.017,\:0.05\}$	1) $\:\lambda\:$ 2) $\:\lambda\:$ and $\:\rho\:$
Scenario 2	RCM-scSCR	$\:({\lambda\:}_{1},\:{\lambda\:}_{2})\in\:\{\left(0.03,\:0.01\right),\:(0.1,\:0.03\left)\right\}\:$ $\:\rho\:=\:\{0.017,\:0.05\}$ $\:\tau\:=\{3,\:5,\:10\}\:$	1) $\:{\lambda\:}_{1}$ and $\:{\lambda\:}_{2}$ 2) $\:{\lambda\:}_{1},\:{\lambda\:}_{2},\:\mathrm{a}\mathrm{n}\text{d }\:\rho\:$

RCM: Reverse catalytic model, scSCR: single change point sero conversion rate, $\:\lambda\:$: Sero conversion rate (SCR), $\:\rho\:$: Sero reversion rate (SRR), $\:{\lambda\:}_{1}$: SCR before the change point, $\:{\lambda\:}_{2}$: SCR after the change point, $\:\tau\:$: Years before sampling.

### Simulations

To identify the optimal allocation of samples across age groups, we conducted Monte-Carlo simulations with 1000 iterations for generating each required dataset in each scenario, given different sample sizes (*N* = 250, 500, 1000, and 2000). For Scenario 1, we varied age-based sampling structures by changing the proportion among five age groups ([1,10], [11, 20], [21, 30], [31, 40], and [41, 50] years of age) from 0 to 100% in increments of 20%, resulting in 126 distributions (Supplementary Fig. 1). For scenario 2, we examined how different sampling structures inform the estimation of SCRs before and after the change point. Individuals born after the change point provide information specific to the post-change SCR, whereas those born before the change point contribute information on cumulative exposure across both periods. Therefore, including both birth cohorts is important for separating the two parameters. Accordingly, the sampling structures were adapted to five age groups (in years of age): [1, $\:\tau\:$], [$\:\tau\:+1$, $\:\tau\:+10$], [$\:\tau\:+11$, $\:\tau\:+20$], [$\:\tau\:+21$, $\:\tau\:+30$], and [$\:\tau\:+31$, $\:\tau\:+40$]. For a given value of τ, these correspond to fixed age intervals (e.g., [1, 10], [11, 20], [21, 30], [31, 40], and [41, 50] when τ = 10). The proportion of the first age group varied from 20 to 80% in increments of 20%, while the proportions of the remaining age groups varied from 0 to 80% in increments of 20%. This allowed us to explore sampling distributions with varying contributions from pre- and post-change cohorts. In total, 69 sampling distributions were considered (Supplementary Fig. 2). In each iteration, we generated a hypothetical dataset of N individuals by sampling ages according to the specified proportions for each age group. Using the age-specific probability of seropositivity based on Eq. (1) or (2), depending on the underlying assumption of the scenario, we then generated the binary outcome of seropositivity for each individual using a binomial distribution.

### Evaluation metrics for parameter estimation

Precision was defined as the variability of the estimator under repeated sampling and quantified as half the length of the interval between the 2.5th and 97.5th percentiles of the estimated parameter obtained from 1,000 simulations for each set of scenarios and assumptions. Because the total sample size and estimation procedure were held constant across scenarios, differences in this measure primarily reflect how alternative age distributions influence the stability of the estimates. Relative precision was then calculated as the precision divided by the true parameter value that generated the corresponding data. The optimal allocations were determined based on the relative precision of SCR estimates obtained under different age distributions. For Scenario 2, the optimal allocations were identified using a joint precision, defined as the sum of the relative precisions of SCR before and after the change point. We also quantified bias and root mean squared error (RMSE) of the estimated SCRs. Bias was defined as the difference between the estimated and true parameter values, averaged across simulations. RMSE was defined as the square root of the mean squared difference between estimated and true values. These metrics were computed using the same pooled distribution of estimates within each group of sampling structures.

As a sub-analysis, we evaluated the impact of imperfect assay performance on the estimation results by assuming both sensitivity and specificity to be 90%. Although antibody measurements obtained using multiplex immunoassays, which are expected to be increasingly used in future serosurveillance studies, have generally been reported to show high diagnostic accuracy, their sensitivity and specificity may not be perfect^21^. To reflect this realistic source of measurement error, we incorporated imperfect classification of serostatus into the simulation framework and examined how the SCR estimates changed under this scenario. Specifically, we incorporated sensitivity (*\:Se*) and specificity (*\:Sp*) into the likelihood function. Let $\:p\left(a\right)$ denote the true probability of seropositivity at age *\:a*, as defined in Eq. (1) or (2). The observed probability of testing seropositive, $\:{p}_{obs}\left(a\right)$, was then modeled as:

$$\:{p}_{obs}\left(a\right)=Se\:\times\:\:p\left(a\right)+(1-Sp)\times\:(1-p(a\left)\right)\:$$

which accounts for both false negatives and false positives. The likelihood was constructed using $\:{p}_{obs}\left(a\right)$, and parameters were estimated under two approaches. In the naive approach, misclassification in the observed serostatus was ignored and the observed data were treated as true. In the adjusted approach, the likelihood explicitly incorporated imperfect sensitivity and specificity to account for misclassification in the observed serostatus. Precision, bias, and MRSE were evaluated under both approaches following the same procedures.

In addition, for Scenario 2, we assessed the statistical power to detect a reduction in SCR using the likelihood ratio test (LRT) to evaluate whether the RCM-scSCR model provided a better fit to the simulated data than a model assuming a stable SCR. A p-value less than 0.05 was considered statistically significant. Statistical power was defined as the proportion of datasets (out of 1000 simulations) in which a statistically significant result was obtained. Furthermore, after calculating power for each combination of parameters and sample sizes, logistic regression models were fitted to the power estimates with sample size included as a covariate. The predicted power curves from the regression models were presented alongside the power estimates from simulations with sample sizes of *N* = 250, 500, 1000, and 2000.

All simulations were performed using Julia (version 1.10.3), and data summarization and figure generation were conducted in R (version 4.3.3).

### Case study using published serological data in Sri Lanka

To illustrate the potential impact of sampling design, we applied the proposed simulation framework to scenarios inspired by a previously published malaria serological study conducted in Sri Lanka in an elimination setting^22^. In the study, the sampled population in Kurunegala district was heavily skewed toward adults, with 86.0% of individuals aged 20 years or older. As a result, relatively wide confidence intervals for the SCR were reported, and no clear evidence of a change in transmission was identified, with the data being adequately described by a model assuming a single force of infection. We assumed a scenario in which a change in transmission had occurred, using the reported point estimates (λ₁ = 0.0026 before the change point, λ₂ = 0.0001 after the change point, and τ = 12 years). Under this assumption, we generated simulated datasets reflecting (i) the original age distribution reported in the study and (ii) alternative age-based sampling structures derived from our optimization framework. For each sampling structure, we generated 1,000 simulated datasets using two total sample sizes: the original sample size reported in the study (*N* = 637) and an increased sample size (*N* = 2,000). Age distributions and serostatus outcomes were generated according to the procedures described above. SCRs before and after the change point were estimated using MLE, and precision and statistical power were quantified as previously defined.

## Results

### Scenario 1: Under stable malaria transmission intensity

#### Estimation performance of SCR across sampling strategies

**Fig. 2:**
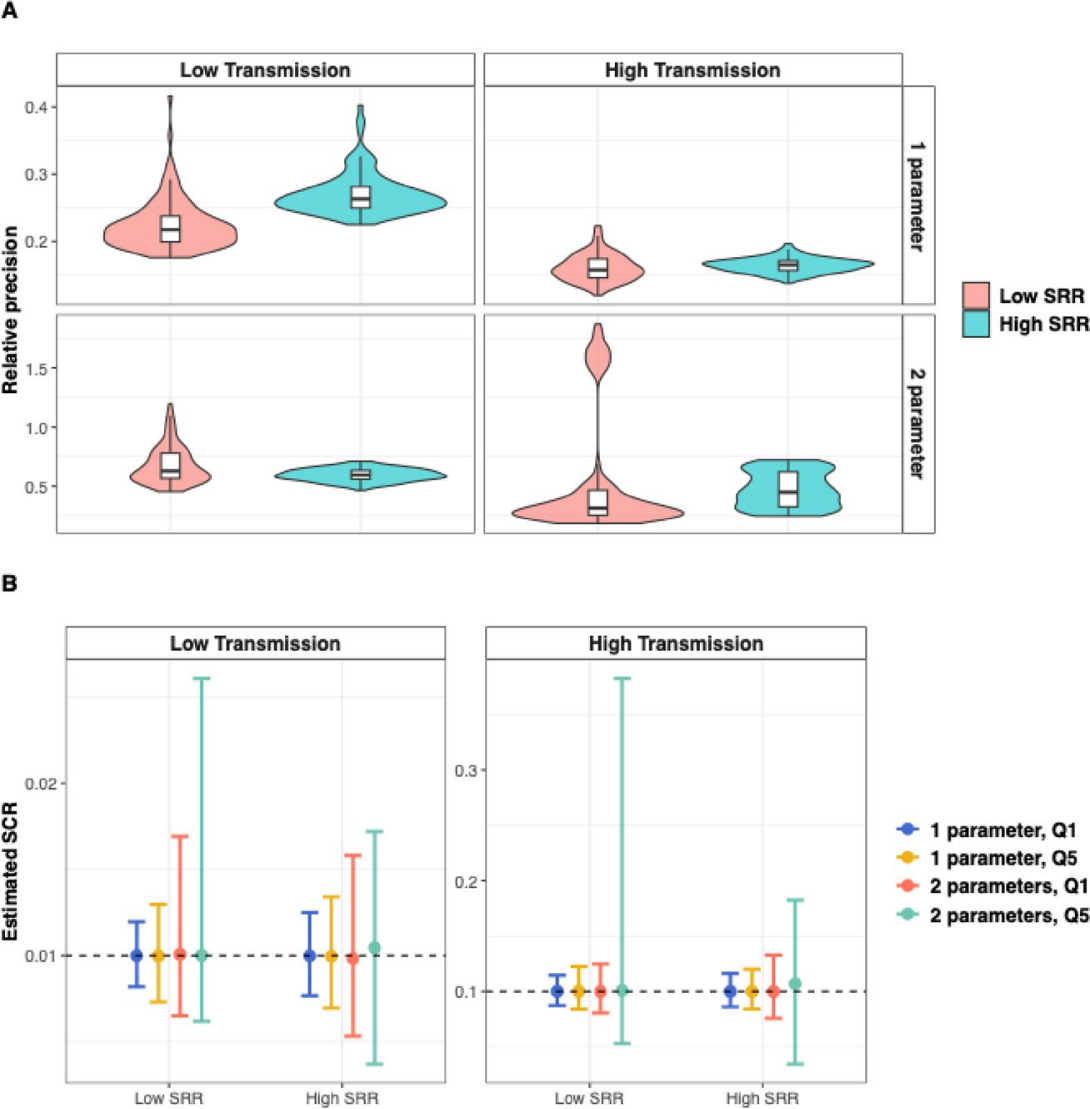
**(A)** Relative precisions and **(B)** empirical 95% intervals of the estimated seroconversion rate (SCR) under a stable SCR scenario with a total sample size of 500. Results are stratified by (1) transmission intensity (SCR = 0.01 or 0.1), (2) seroreversion rate (SRR = 0.017 or 0.05), and (3) the number of parameters estimated (1 parameter: SCR only; 2 parameters: SCR and SRR). Panel B is further stratified by the age structures that yielded the top 20% relative precision (Q1) and the bottom 20% relative precision (Q5). Each dot in Panel B represents the median estimated SCR within each group. Each dot in Panels B represents the median estimated SCR, and error bars indicate the empirical 95% interval based on the pooled distribution of estimates across simulations and sampling structures within each group. Note that the y-axis scales differ across panels to reflect differences in parameter magnitude and model specification.

We evaluated the relative precision of estimated SCR across 126 age-based sampling structures under varying assumptions about the SCR, SRR, and the number of parameters estimated. Relative precision was generally lower in scenarios with a low SCR (i.e., low transmission setting), a high SRR, and when both SCR and SRR were jointly estimated (Fig. 2A). To further examine the impact of age-based sampling structures on SCR estimation precision, we examined the empirical 95% intervals of SCR estimates for age structures that yielded the top 20% (Q1) and bottom 20% (Q5) of relative precision in each combination of parameters (Fig. 2B). Across all categories, SCR estimates were unbiased. However, the sampling structure had a marked influence on precision. In particular, under conditions of low SRR and when SCR and SRR were jointly estimated, Q1 age allocations improved the relative precision compared to Q5 by 1.9-fold and 7.4-fold in low- and high-transmission settings, respectively.

Supplementary Table 1 provides a detailed summary of estimation performance across all groups (Q1–Q5), including median, relative precision, bias, RMSE. Median estimates were close to the true values across all groups and settings, while Q1 consistently outperformed Q5 across all metrics. Under imperfect assay conditions assuming 90% sensitivity and specificity, the naive approach that ignores misclassification led to systematic bias. In low-transmission settings (SCR = 0.01), SCR was overestimated, whereas in high-transmission settings (SCR = 0.1), SCR was underestimated. For one-parameter estimation, the magnitude of bias increased as precision decreased, with larger bias observed in Q5 compared to Q1. In contrast, when both SCR and SRR were jointly estimated, substantial bias was observed regardless of the precision ranking (Supplementary Table 2). When misclassification was accounted for using a likelihood that incorporates sensitivity and specificity, bias was substantially reduced across all settings, with lower bias observed in sampling structures with higher precision (Supplementary Table 3).

#### Optimal age allocations

**Fig. 3:**
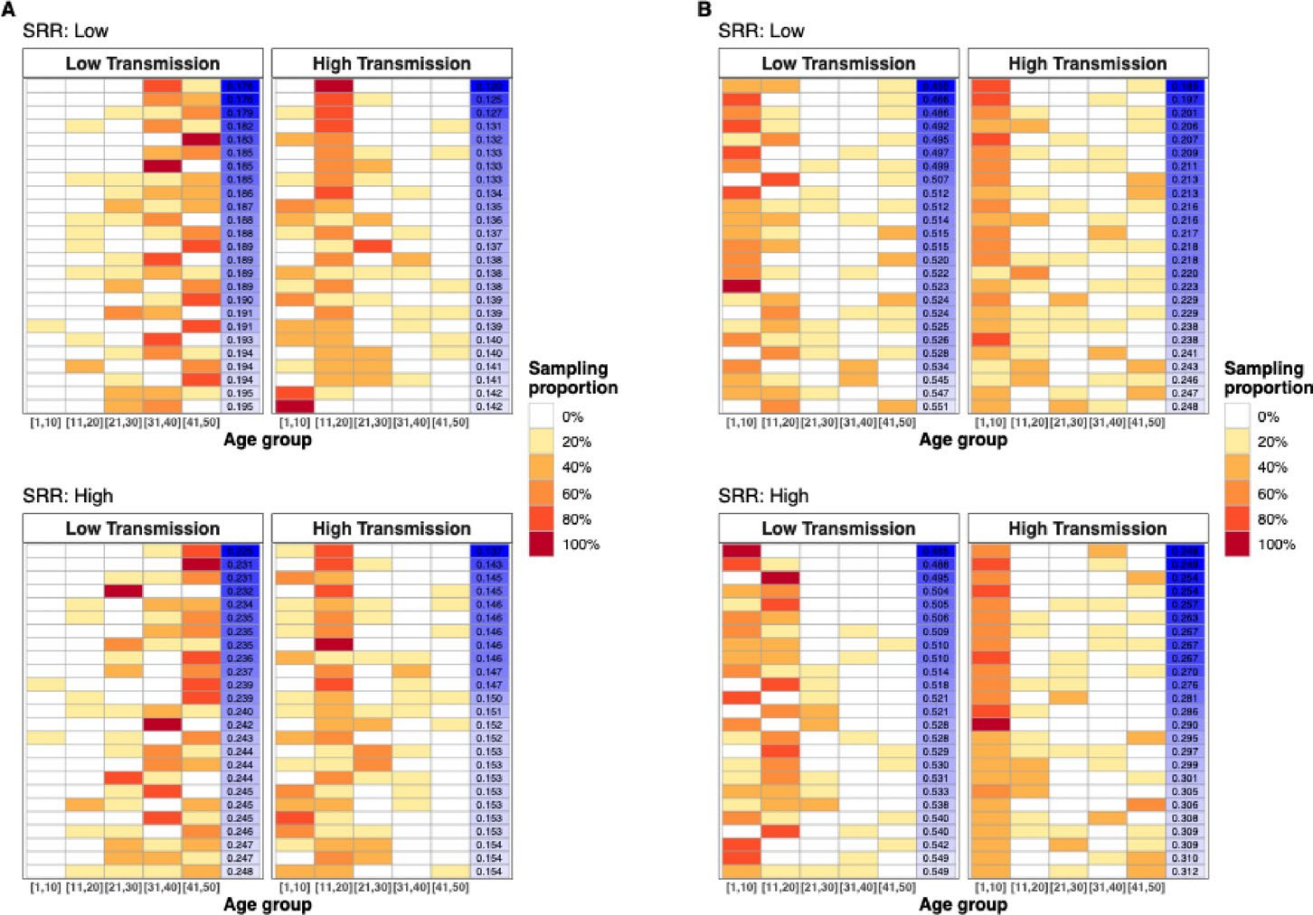
Age-based sampling structures that yielded the top 20% relative precision under the stable malaria transmission intensity scenario with a total sample size of 500. Panels show results for **(A)** estimation of the seroconversion rate (SCR) only, and **(B)** joint estimation of SCR and the seroreversion rate (SRR). Each row represents a distinct sampling strategy, ordered by relative precision, with lower values indicating higher precision (i.e., highest precision at the top). Columns indicate age groups, and colours represent the proportion of the total sample allocated to each age group. The additional column on the right shows the relative precision associated with each sampling strategy.

Optimal age-based sampling structures also varied depending on the number of parameters estimated. When estimating only the SCR, optimal sampling was highly sensitive to the underlying transmission intensity but was largely unaffected by the SRR. Sampling skewed toward older groups was favored in low transmission settings, whereas sampling skewed toward young children was more effective in high transmission settings (Fig. 3A). Conversely, the opposite sampling strategies—sampling young children in low transmission settings and adults in high transmission settings—resulted in the lowest precision for estimating SCR (Supplementary Fig. 3 A). When both the SCR and SRR were estimated jointly, the optimal sampling structures were more consistent across transmission settings. Sampling strategies that prioritized younger children yielded the highest precision, whereas those focused on older adults resulted in the poorest estimation precision (Fig. 3B, Supplementary Fig. 3B). These trends remained consistent when the total sample size increased from 500 to 1,000 (Supplementary Figs. 4 and 5).

### Scenario 2: Under a single change point in malaria transmission intensity

#### Estimation performance of SCR before and after the change point across sampling strategies

**Fig. 4:**
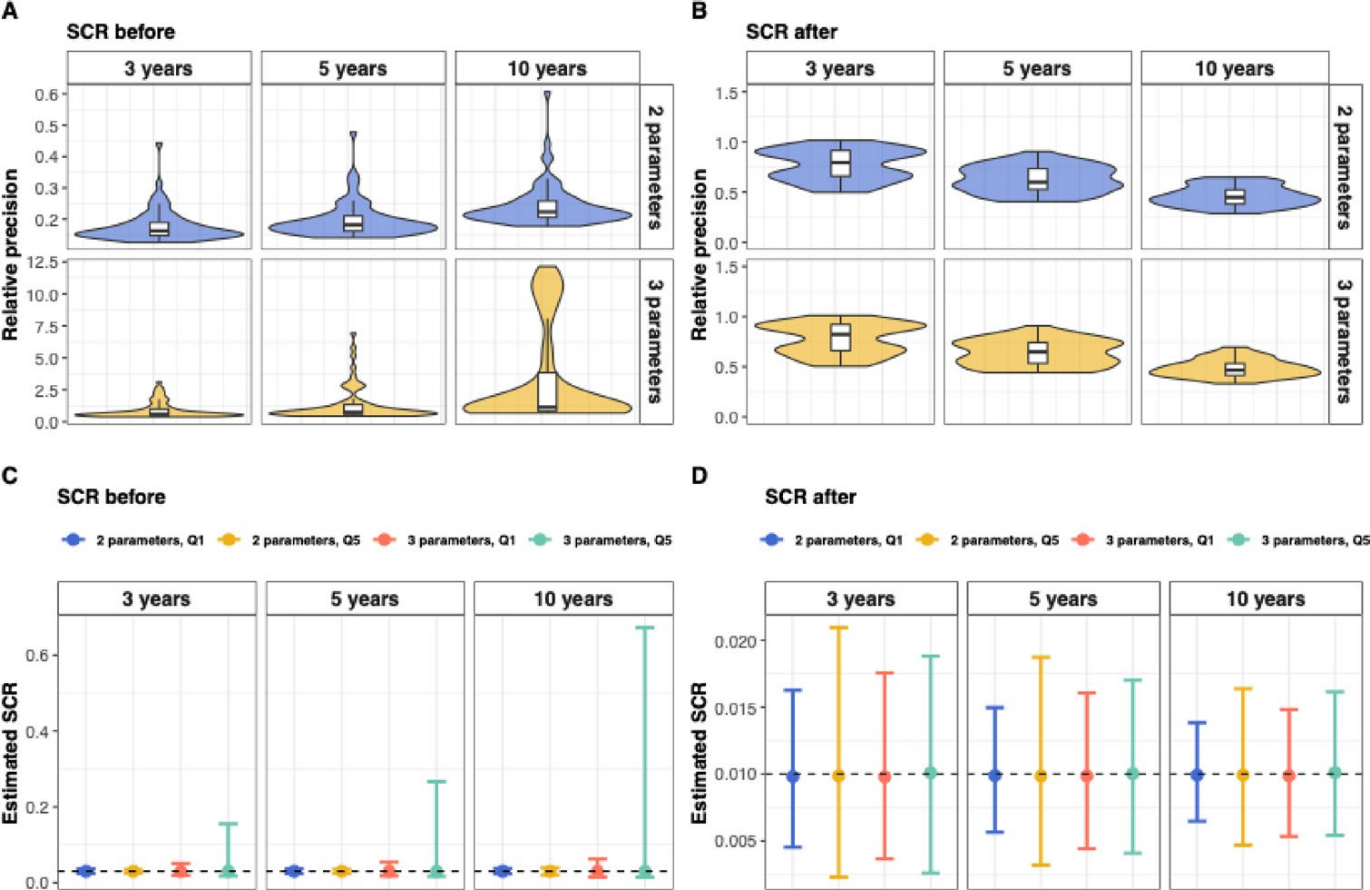
Relative precisions **(A and B)** and empirical 95% intervals **(C and D)** of the estimated seroconversion rates (SCRs) before **(A and C)** and after **(B and D)** a single change point in malaria transmission intensity. The analysis assumed a total sample size of 1,000 with low underlying SCRs (SCR = 0.03 before and 0.01 after the change point) and a low seroreversion rate (SRR = 0.017). Results are stratified by (1) the time since the change point ($\:\tau\:$ = 3, 5, and 10 years, representing the time between the change point and data collection) and (2) the number of parameters estimated (2 parameter: SCRs before and after the change point; 3 parameters: SCRs and SRR). Panels C and D are further stratified by the age structures that yielded the top 20% joint precision (Q1) and the bottom 20% joint precision (Q5). Each dot in Panels C and D represents the median estimated SCR, and error bars indicate the empirical 95% interval based on the pooled distribution of estimates across simulations and sampling structures within each group. Note that the y-axis scales differ across panels to reflect differences in parameter magnitude and model specification.

Figure 4 shows the precision and empirical 95% intervals of the estimated SCRs before and after the change point, assuming a total sample size of 1,000, low transmission settings (SCR = 0.03 before and 0.01 after the change point), and a low SRR (0.017). The precision of the SCR estimate before the change point declined as the number of years since the change point increased, while the precision of the SCR after the change point improved over time. When comparing models that estimated only two parameters (SCRs) with those that jointly estimated three parameters (SCRs and SRR), there was little difference in the precision of the SCR estimates after the change point. However, the precision of the SCR estimates before the change point deteriorated when three parameters were estimated. Notably, even under the three-parameter estimation settings, age allocations classified as Q1 yielded relatively high precision and narrow 95% intervals for the SCR before the change point. In contrast, Q5 age allocations resulted in substantially wider 95% intervals, particularly when the change point occurred 10 years prior to the sampling (Fig. 3C). While high transmission settings (SCR = 0.1 before and 0.03 after the change point) showed better overall precision compared to lower transmission settings, the above-mentioned trends remained consistent (Supplementary Fig. 6). Supplementary Table 4 provides a detailed summary of estimation performance for the SCRs across all groups (Q1–Q5), including median estimates, relative precision, bias, and root mean squared error RMSE. Median estimates were close to the true values across all groups and settings, with bias generally close to zero. In the two-parameter estimation settings, when the time since the change point was short (τ = 3 and 5 years), the relative precision of the pre-change SCR was higher in Q2–Q5 than in Q1. However, the overall magnitude of precision for the pre-change SCR was relatively high and the differences across sampling structures were modest, particularly compared with the post-change SCR. In all other settings, Q1 consistently achieved the highest relative precision across parameters. Under imperfect assay conditions assuming 90% sensitivity and specificity, the naive approach that ignores misclassification led to systematic bias. In the low-transmission setting (SCR 0.03 before and 0.01 after the change point), the pre-change SCR was underestimated, whereas the post-change SCR was overestimated (Supplementary Table 5). When misclassification was accounted for using a likelihood that incorporates sensitivity and specificity, bias was markedly reduced across all settings (Supplementary Table 6). However, relative precision was substantially lower compared with the scenario assuming perfect assay performance.

#### Optimal age allocations

**Fig. 5:**
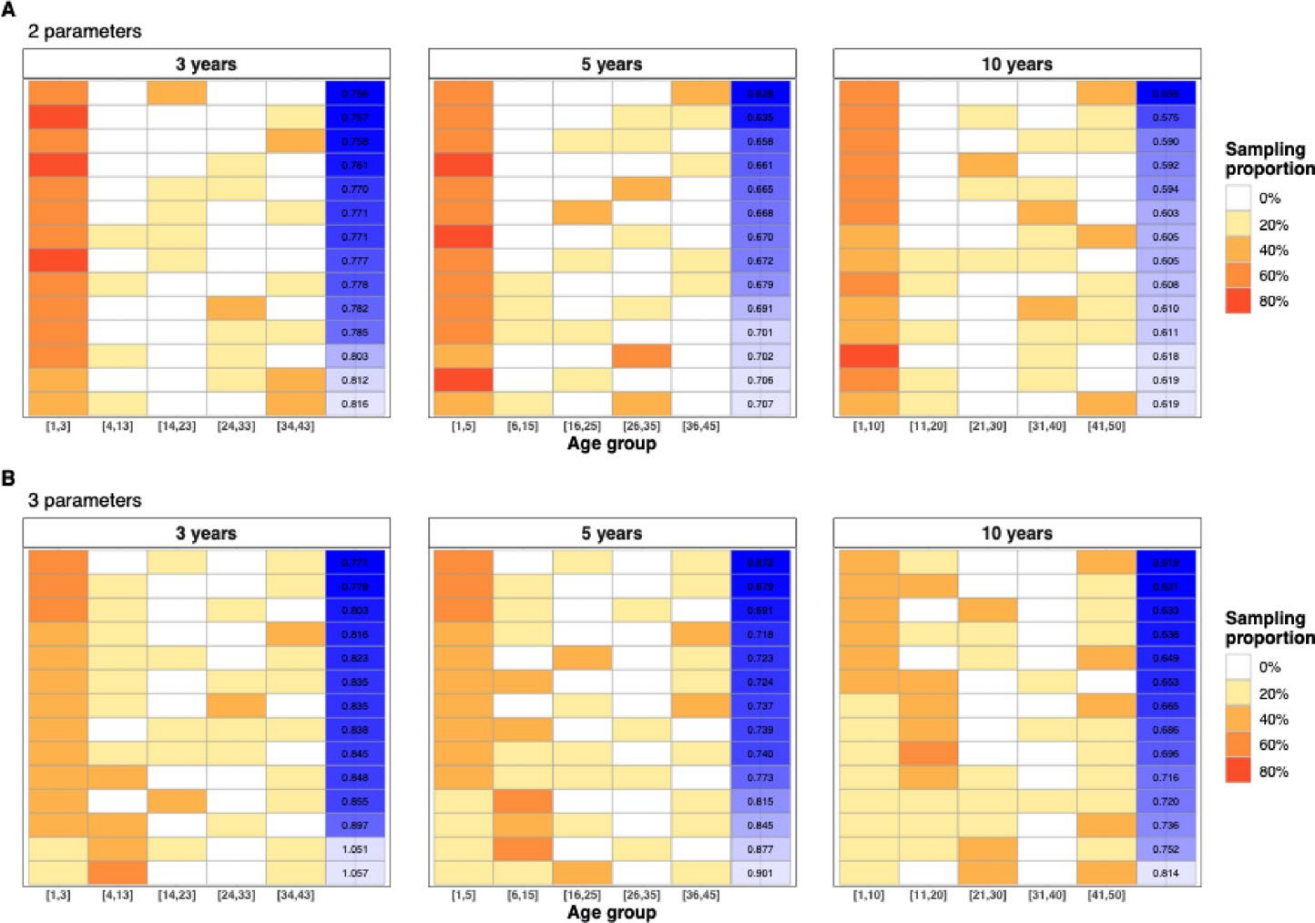
Age-based sampling structures that yielded the top 20% joint precision under a single change point in malaria transmission intensity scenario. The analysis assumed a total sample size of 1,000 with low underlying SCRs (SCR = 0.03 before and 0.01 after the change point) and a low seroreversion rate (SRR = 0.017). Results were stratified by the time since the change point (3, 5, and 10 years). **(A)** Only the SCR before and after the change point were estimated. **(B)** The SCRs and the seroreversion rate (SRR) were jointly estimated. Each row represents a distinct sampling strategy, ordered by relative precision, with lower values indicating higher precision (i.e., highest precision at the top). Columns indicate age groups, and colours represent the proportion of the total sample allocated to each age group. The additional column on the right shows the relative precision associated with each sampling strategy.

Figure 5 shows the optimal age allocations for maximizing the joint precision of SCR estimates before and after the change point under low transmission settings (based on the same assumption as in Fig. 4). When estimating two parameters, higher precision was achieved by sampling at least 40% of individuals from the age group born after the change point. In contrast, when estimating three parameters, a more balanced age allocation was preferred. This involved slightly reducing the proportion of children born after the change point to 20–60% and increasing sampling from older age groups. Sampling only 20% from the first age group in the two-parameter estimation scenario and sampling exclusively from the first, fourth, and fifth age groups in the three-parameter estimation scenario resulted in the poorest joint precision of SCR estimates (Supplementary Fig. 7). Under high transmission settings (SCR = 0.1 before and 0.03 after the change point), the optimal allocation was largely consistent with that observed under low transmission settings (Supplementary Figs. 8 and 9).

#### Statistical power to detect a change in SCR

**Fig. 6:**
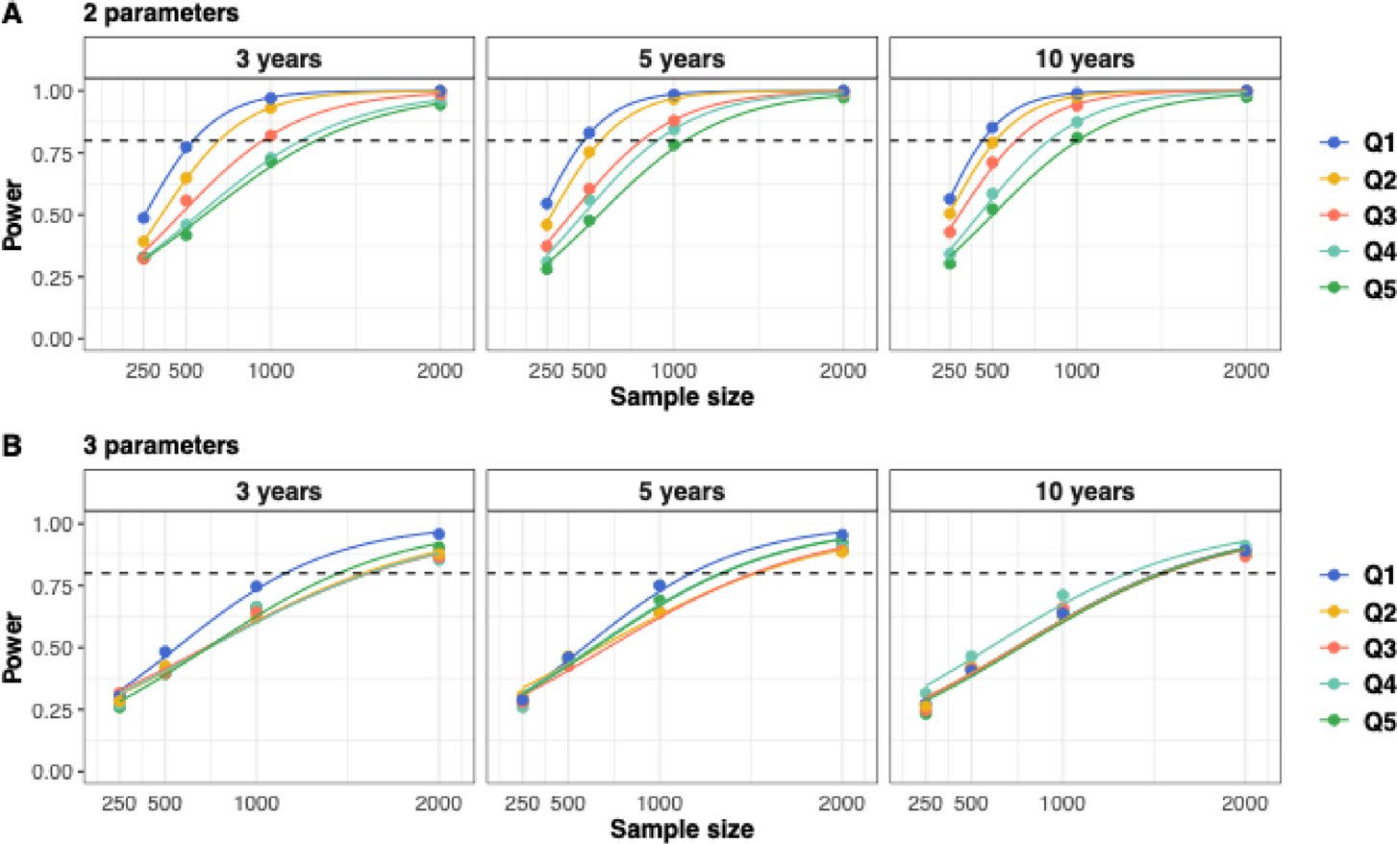
Statistical power to detect a change in seroconversion rate in low transmission settings (SCR, 0.03 before and 0.01 after the change point). Solid lines represent the fitted logistic functions applied to the corresponding simulated results (filled circles), stratified by the quintiles of joint precision (Q1–Q5). **(A)** Only the SCR before and after the change point were estimated. **(B)** The SCRs and the seroreversion rate (SRR) were jointly estimated.

Age allocations that yielded higher joint precision also resulted in higher statistical power for the two-parameter estimation in both low and high transmission settings (Fig. 6A and Supplementary Fig. 10 A). However, for the three-parameter estimation, optimal age allocations based on joint precision did not consistently lead to improved power, and the power was lower compared to that of the two-parameter estimation (Fig. 6B and Supplementary Fig. 10B).

### Case study in *Sri Lanka*

**Fig. 7:**
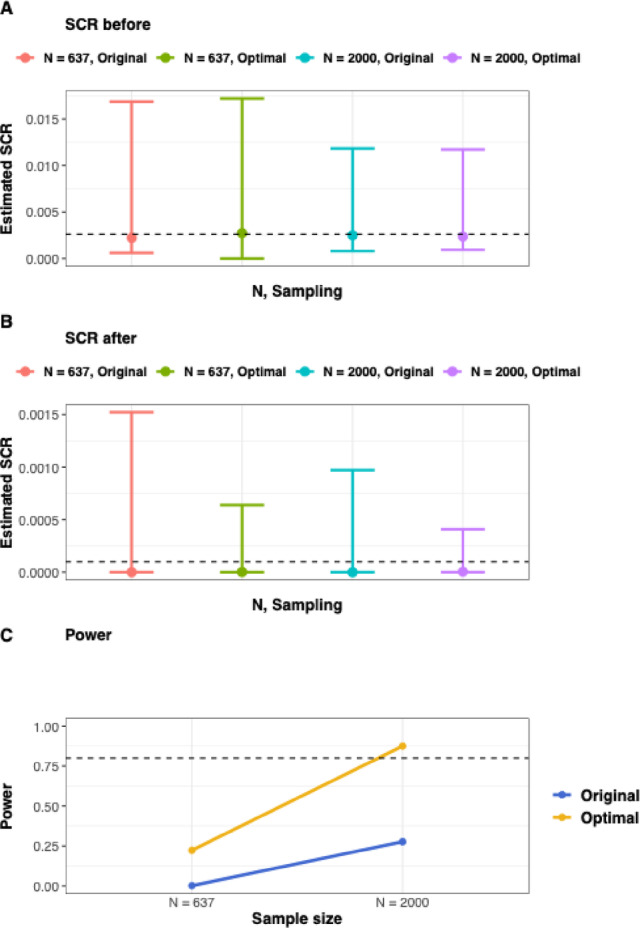
Comparison of estimation precision and power between the original sampling reported by Dewasurendra et al. and the optimized sampling design. **(A)** Estimated seroconversion rates (SCRs) before the change point. **(B)** Estimated SCRs after the change point. **(C)** Power to detect a change in transmission using a likelihood ratio test. Results are shown for two total sample sizes (*N* = 637 and *N* = 2,000) under both the original sampling structure and the optimized sampling design. In Panels A and B, points represent median estimates and error bars indicate empirical 95% intervals based on the distribution of estimates across simulations.

The age distribution of the original study population was heavily skewed towards older individuals, whereas the optimized sampling structures allocated a larger proportion of samples to younger age groups (Table 2). Under the original sampling design, the estimated SCRs showed relatively wide precisions, particularly for the post-change SCR. Under the optimized sampling design, the precision of the post-change SCR improved, while the precision of the pre-change SCR remained similar. The power to detect a change in transmission increased from approximately 30% under the original sampling structure to 87% under the optimized design when the total sample size was increased to 2,000 (Fig. 7).

**Table 2:** Comparison of age distributions between the original study conducted by Dewasurendra et al. and the optimized sampling design used in this study.

	Original study (Dewasurendra et al.)	Optimal sampling
**Age distribution**		
1–5 years	3.1%	20.0%
6–10 years	2.0%	20.0%
11–20 years	8.7%	40.0%
> 20 years	86.0%	20.0%

### Shiny application

Supplementary Fig. 11 displays the user interface and output of the Shiny application. By specifying the scenario, values for SCR and SRR, the change point (for the change-in-SCR scenario), and the number of parameters to be estimated, the user can obtain the 95% CI for the SCR(s) along with the age-based sampling structures that yield the highest and lowest precision.

## Discussion

This study identified optimal age-based sampling strategies using the RCM and scRCM models, both of which are widely employed in malaria seroepidemiological research. In both models, the results indicate that the optimal age allocation of samples is highly dependent on whether the SRR is known in the study setting. When the SRR is known, optimal sampling is strongly influenced by the transmission intensity, or the values of SCR. In contrast, when estimating both the SCR and SRR, the optimal allocation is less sensitive to the SCR. Careful consideration of sampling design is critical to enhancing study efficiency, particularly in settings with limited personnel or financial resources.

When the SRR is known and transmission intensity is stable, allocating samples toward older adults in low-transmission settings and toward children in high-transmission settings yields more precise estimates of the SCR. Nevertheless, even the least efficient sampling strategies under the known-SRR settings (i.e., those in the bottom 20% for precision) can yield better precision compared to the most efficient sampling strategies under the unknown-SRR settings, where SCR and SRR must be jointly estimated. This suggests that the practical advantage of employing highly skewed sampling schemes may be limited. In cases where the SRR is unknown, the least precise age-based sampling structures (Q5) showed substantially poorer performance under low SRR conditions than under high SRR conditions. Specifically, the relative precision for Q5 was 0.9954 when SRR was assumed to be 0.017 (Supplementary Table 1 A), compared with 0.6750 when SRR was assumed to be 0.05 (Supplementary Table 1B). These less effective sampling structures were typically skewed toward older age groups, with few samples collected from young children (Supplementary Fig. 3B). A representative example is sampling exclusively from individuals over 20 years of age. As shown in Fig. 1A, seroprevalence patterns for individuals under age 15 are more distinct across SCR–SRR combinations, whereas substantial overlap occurs at older ages. For instance, the seroprevalence between ages 20 and 30 for SCR 0.017 and SRR 0.03 overlaps with that for SCR 0.034 and SRR 0.09. This overlap explains why sampling only from adults results in lower precision when SRR is unknown. Given that joint estimation of SCR and SRR is more common in practice, prioritizing sampling among children, with adjustments based on real-world field conditions, may provide a more feasible and efficient approach to study implementation, regardless of the transmission intensity.

By changing transmission settings over time, we found that higher precision for both the pre- and post-change SCRs could be achieved—even when SRR was unknown—by optimizing the age-based sample allocation. These optimal sampling structures for such joint estimation of SCRs and SRR were relatively insensitive to the underlying transmission intensity and tended to distribute samples evenly across age groups born approximately 0–20 years and 20–40 years before the change point. In addition to improving estimation precision, these sampling structures also contributed to higher statistical power for detecting changes in the SCR when the SRR was known. However, when the SRR was unknown, optimal age allocations based on joint precision did not consistently lead to improved power. It is important to note, however, that this sampling strategy was designed to improve the precision of both pre- and post-change SCR estimates. This is relevant because such analyses often aim to assess the impact of interventions or changes in risk factors introduced around the change point^7,14^. The small difference in the precision of post-change SCR estimates between the two-parameter and three-parameter models can be attributed to the limited information that seroprevalence among individuals born after the change point provides about the SRR. This is illustrated in Fig. 1B, which shows clear separation in seroprevalence patterns across different combinations of SCR and SRR values (SCR: 0.01, 0.02, 0.03; SRR: 0.01, 0.03, 0.06, 0.09) for those born after the change point. In contrast, substantial overlap is observed among the pre-change age groups, where similar seroprevalence patterns can arise from multiple combinations of SCR and SRR values.

In both scenarios, bias was negligible across all sampling structures when perfect assay performance was assumed. In contrast, when using observed data generated under imperfect assay conditions with 90% sensitivity and specificity, substantial and systematic bias was observed in the estimated SCRs (Supplementary Tables 2 and 5). The assumed level of assay performance (90% sensitivity and specificity) represents a conservative scenario, and in practice, diagnostic accuracy is likely to fall between 90% and 100%^21^. These findings suggest that caution is warranted when interpreting SCR estimates based solely on observed serological data. Incorporating plausible values of sensitivity and specificity into the likelihood function, as implemented in this study, may provide more reliable estimates of SCR (Supplementary Tables 3 and 6). Under such conditions, the optimal age-based sampling strategies identified in this study remained advantageous in terms of estimation performance.

Across both scenarios, the joint estimating SCR(s) and SRR led to a reduction in the precision of SCR estimates, along with a notable decline in the statistical power to detect changes in SCR. To address these challenges, future studies may benefit from employing Bayesian approaches that incorporate informative prior distributions for SRR, drawing on previously reported values ranging from 0.01 to 0.05^7,17^. Under such approaches, the resulting optimal age-based sampling allocations are expected to more closely approximate those identified in the present study under models estimating SCR(s) alone.

The case study based on the data from Dewasurendra et al. illustrates how age-based sampling design can substantially influence both the precision of SCR estimates and the ability to detect changes in transmission. In the study by Dewasurendra et al., the relatively high precision observed for the pre-change SCR is likely attributable to the sampling design being heavily skewed toward adults aged over 20 years. However, this came at the cost of substantially reduced precision for the post-change SCR, reflecting the limited representation of younger individuals who are more informative of recent transmission following the change point. Furthermore, in the sampling structure used in Dewasurendra et al., statistical power remained limited even when the sample size was increased, indicating that sample size alone may not be sufficient to ensure adequate power. In studies where a single change point is not supported by LRT, the absence of statistical significance may not necessarily indicate a true lack of change in transmission. Rather, it may reflect insufficient statistical power due to suboptimal age-based sampling, highlighting the importance of considering both sample size and sampling design when interpreting evidence for changes in transmission intensity. It is worth noting that, across all previous studies we identified that applied stable SCR models, age sampling was generally well balanced. As a result, we did not identify studies in which the type of optimization proposed here led to substantial gains in precision. Nonetheless, these findings underscore the importance of avoiding highly skewed sampling structures in practice.

This study has several limitations. First, we explored a limited range of SCR and SRR values, focusing specifically on MSP1, and did not consider other antigens associated with longer-lived antibody responses, such as *P.falciparum* apical membrane antigen-1 (AMA1)^23,24^ and *P. falciparum* glutamate rich protein (*Pf*GLURP.R2)^25,26^. These alternative antigens may reflect different durations or patterns of exposure and could be used to complement or expand the current analysis. Alternative antigen combinations and parameter ranges can be explored through the Shiny application. Second, the RCM relies on simplifying assumptions, including homogeneous transmission and fixed SCR and SRR, with individuals transitioning between seronegative and seropositive states accordingly. In real-world settings, however, malaria transmission is often heterogeneous across space and time, and population movement between areas with different transmission intensities may further complicate exposure histories. These factors are not explicitly accounted for in the study and may influence both SCR estimation and the performance of sampling strategies. Future work extending this approach to incorporate transmission heterogeneity, migration, and more complex dynamics, such as gradual transitions or multiple change points, would be valuable. In addition, the SRR may vary with the frequency of antigenic exposure. The superinfection model extends the RCM by incorporating multiple seropositive states to reflect repeated infections^27^. Although biologically plausible, previous studies have demonstrated that incorporating superinfection yields minimal improvements in SCR estimation, particularly in low-transmission settings^7,27^. Third, we employed MLE rather than a Bayesian approach, which would allow the incorporation of prior information and more flexible model extensions. This choice was primarily motivated by practical considerations, as MLE can be implemented using relatively straightforward optimization procedures. We acknowledge that contemporary software has made Bayesian approaches increasingly accessible; however, our aim was to focus on a framework that can be readily implemented and interpreted in applied settings. Nevertheless, because MLE does not incorporate prior information, it occasionally produced extreme SCR estimates when jointly estimating SRR. In contrast, a Bayesian framework could provide natural regularization of parameter estimates, constraining them to plausible ranges without requiring explicit penalties in the likelihood. Fourth, the number of age groups used to determine the optimal allocation of a fixed sample size was deliberately restricted to reduce the computational burden. Allowing for a large number of finely stratified age groups would have dramatically increased the number of possible allocation combinations, making the optimization process computationally intensive and potentially infeasible for routine use. While this simplification facilitated analysis, it may have limited our ability to detect more granular patterns in age-based sampling structures.

## Conclusions

Careful selection of age-based sampling structures can improve the precision of SCR estimates in malaria seroepidemiology. In settings where the SRR is uncertain, sampling strategies that include younger age groups and maintain a balanced age distribution are likely to provide more robust estimates. When estimating SCR from a single cross-sectional serological survey, particular attention should be paid to the distribution of samples across age groups to ensure acceptable levels of precision.

## Supplementary Information

Below is the link to the electronic supplementary material.

Supplementary Material 1

## Data Availability

Replication codes are available on GitHub (https://github.com/KoKYura/malaria_seroepi_sampling).
