# Supplementary material for: Optimizing age-structured sampling for estimating the seroconversion rate in malaria seroepidemiology: a simulation study
Source: Sci Rep. 2026 May 30;16:24684. doi: 10.1038/s41598-026-55736-x (PMC13454486; doi:10.1038/s41598-026-55736-x)
Supplement: Supplementary file 1 [file 41598_2026_55736_MOESM1_ESM.docx]

**Optimizing Age-Structured Sampling for Estimating the Seroconversion Rate in Malaria Seroepidemiology: A Simulation Study**

**Authors**

Yura K Ko^1,2^, Shilei Li^1^, Tom Britton^3^, Wataru Kagaya^4^, Akira Kaneko^1,5^

^1^Department of Microbiology, Tumor and Cell Biology (MTC), Karolinska Institutet, Sweden

^2^Department of Vector Ecology and Environment, Institute of Tropical Medicine (NEKKEN), Nagasaki University, Japan

^3^Department of Mathematics, Stockholm University, Sweden

^4^Department of Ecoepidemiology, Institute of Tropical Medicine (NEKKEN), Nagasaki University, Japan

^5^Department of Virology and Parasitology, Graduate School of Medicine, Osaka Metropolitan University, Japan.

Table of Contents

[Instructions for the shiny application	3](#_Toc227576947)

[Supplementary Tables	5](#_Toc227576961)

[Supplementary Table 1.	5](#_Toc227576962)

[Supplementary Table 2.	6](#_Toc227576963)

[Supplementary Table 3.	7](#_Toc227576964)

[Supplementary Table 4.	8](#_Toc227576965)

[Supplementary Table 5.	10](#_Toc227576966)

[Supplementary Table 6.	12](#_Toc227576967)

[Supplementary Figures	14](#_Toc227576968)

[Supplementary Figure 1	14](#_Toc227576969)

[Supplementary Figure 2	15](#_Toc227576970)

[Supplementary Figure 3	16](#_Toc227576971)

[Supplementary Figure 4	17](#_Toc227576972)

[Supplementary Figure 5	18](#_Toc227576973)

[Supplementary Figure 6	19](#_Toc227576974)

[Supplementary Figure 7	20](#_Toc227576975)

[Supplementary Figure 8	21](#_Toc227576976)

[Supplementary Figure 9	22](#_Toc227576977)

[Supplementary Figure 10	23](#_Toc227576978)

[Supplementary Figure 11	24](#_Toc227576979)

# **Instructions for the shiny application**

To run the shiny application locally on your computer, follow the steps below:

0. Install R, Julia, and RStudio:
If you have not already installed R, Julia, and RStudio, download and install them.

- **R**: <https://cran.r-project.org/>
- **Julia**: <https://julialang.org/>
- **RStudio**: <https://posit.co/download/rstudio-desktop/>

1. Download the shiny app files:
You can download all the necessary files from here (<https://github.com/KoKYura/malaria_seroepi_sampling>)

2. Install required R packages:
Open Rstudio and install the necessary packages by running the following commands in the R console.

install.packages("shiny")
install.packages("shinydashboard")
install.packages("showtext")
install.packages("DT")
install.packages("JuliaCall")

3. Configure Julia from R:
After installing the R packages, set up the Julia environment through JuliaCall by running:

library(JuliaCall)
julia_setup()

If Julia is not detected automatically, specify the path to your Julia installation manually within julia_setup().

The application additionally depends on the following Julia packages: **[Distributions, DataFrames, JLD2, Random, Optim]**. These should be installed in the local Julia environment before launching the app by running:

4. Run the application:
Open the “app.R” file in Rstudio, and launch the application either clicking the “Run App” button in Rstudio or running the following command in the R console:

shiny::runApp()

5. Select a scenario from the left panel:
Choose the simulation scenario based on your interest: 1) Stable SCR or 2) Change in SCR.

6. Set parameters and run simulations:
Modify the following parameters:

For the Stable SCR scenario,

1. Number of samples, 2. Seroconversion rate (SCR), 3. Seroreversion rate (SRR), 4. Estimating parameter (SCR only, SCR and SRR)

For the Change in SCR scenario,

1. Number of samples, 2. SCR before the change point, 3. SCR after the change point, 4. SRR, 5. Change point (Years), 6. Estimating parameter (SCRs only, SCRs and SRR)

After setting all parameters, click “Run” to start the simulation. Once the simulation is complete, you will obtain the the 95%CI for the SCR(s) along with the age-based sampling structures that yield the highest and lowest precision.

# Supplementary Tables

Supplementary Table 1. Summary of estimation performance for the seroconversion rate (SCR) across sampling strategies grouped by relative precision (Q1–Q5) under a stable SCR scenario with a total sample size of 500. Q1 represents the top 20% of sampling structures with the highest precision, and Q5 the lowest. Median estimates, relative precision, bias, and root mean squared error (RMSE) are calculated from the pooled distribution of estimates across simulations and sampling structures within each group, and are reported for both one-parameter and two-parameter models. Results are shown for four combinations of SCR and seroreversion rate (SRR): (A) SCR = 0.01 and SRR = 0.017, (B) SCR = 0.01 and SRR = 0.05, (C) SCR = 0.1 and SRR = 0.017, and (D) SCR = 0.1 and SRR = 0.05.

| **(A)** |  |  |  |  |  |  |  |  |
| --- | --- | --- | --- | --- | --- | --- | --- | --- |
|  | **1 parameter estimation** | | | | **2 parameters estimation** | | | |
|  | **Median** | **Relative Precision** | **Bias** | **RMSE** | **Median** | **Relative Precision** | **Bias** | **RMSE** |
| Q1 | 0.0100 | 0.1889 | 0.0000 | 0.0010 | 0.0101 | 0.5210 | 0.0005 | 0.0028 |
| Q2 | 0.0100 | 0.2028 | 0.0000 | 0.0010 | 0.0100 | 0.5785 | 0.0007 | 0.0031 |
| Q3 | 0.0100 | 0.2183 | 0.0000 | 0.0011 | 0.0100 | 0.6392 | 0.0009 | 0.0035 |
| Q4 | 0.0100 | 0.2332 | 0.0000 | 0.0012 | 0.0100 | 0.7444 | 0.0012 | 0.0043 |
| Q5 | 0.0100 | 0.2830 | 0.0000 | 0.0014 | 0.0100 | 0.9954 | 0.0022 | 0.0063 |
|  |  |  |  |  |  |  |  |  |
| **(B)** |  |  |  |  |  |  |  |  |
|  | **1 parameter estimation** | | | | **2 parameters estimation** | | | |
|  | **Median** | **Relative Precision** | **Bias** | **RMSE** | **Median** | **Relative Precision** | **Bias** | **RMSE** |
| Q1 | 0.0100 | 0.2419 | 0.0000 | 0.0012 | 0.0098 | 0.5246 | 0.0000 | 0.0028 |
| Q2 | 0.0100 | 0.2543 | 0.0000 | 0.0013 | 0.0099 | 0.5691 | 0.0000 | 0.0032 |
| Q3 | 0.0100 | 0.2656 | 0.0000 | 0.0014 | 0.0100 | 0.5970 | 0.0000 | 0.0033 |
| Q4 | 0.0100 | 0.2777 | 0.0000 | 0.0014 | 0.0101 | 0.6304 | 0.0000 | 0.0036 |
| Q5 | 0.0100 | 0.3227 | 0.0000 | 0.0016 | 0.0104 | 0.6750 | 0.0001 | 0.0040 |
|  |  |  |  |  |  |  |  |  |
| **(C)** |  |  |  |  |  |  |  |  |
|  | **1 parameter estimation** | | | | **2 parameters estimation** | | | |
|  | **Median** | **Relative Precision** | **Bias** | **RMSE** | **Median** | **Relative Precision** | **Bias** | **RMSE** |
| Q1 | 0.1001 | 0.1366 | 0.0003 | 0.0070 | 0.0998 | 0.2216 | 0.0006 | 0.0114 |
| Q2 | 0.1000 | 0.1497 | 0.0003 | 0.0076 | 0.0999 | 0.2657 | 0.0012 | 0.0137 |
| Q3 | 0.1001 | 0.1585 | 0.0005 | 0.0081 | 0.1001 | 0.3146 | 0.0021 | 0.0169 |
| Q4 | 0.1000 | 0.1703 | 0.0006 | 0.0087 | 0.0999 | 0.4193 | 0.0037 | 0.0253 |
| Q5 | 0.1001 | 0.1926 | 0.0009 | 0.0098 | 0.1009 | 1.6462 | 0.0469 | 0.1139 |
|  |  |  |  |  |  |  |  |  |
| **(D)** |  |  |  |  |  |  |  |  |
|  | **1 parameter estimation** | | | | **2 parameters estimation** | | | |
|  | **Median** | **Relative Precision** | **Bias** | **RMSE** | **Median** | **Relative Precision** | **Bias** | **RMSE** |
| Q1 | 0.1000 | 0.1507 | 0.0003 | 0.0077 | 0.0999 | 0.2852 | 0.0011 | 0.0148 |
| Q2 | 0.1001 | 0.1590 | 0.0004 | 0.0082 | 0.1000 | 0.3718 | 0.0022 | 0.0188 |
| Q3 | 0.1000 | 0.1654 | 0.0004 | 0.0085 | 0.1002 | 0.4638 | 0.0034 | 0.0238 |
| Q4 | 0.1001 | 0.1706 | 0.0005 | 0.0087 | 0.1003 | 0.5859 | 0.0073 | 0.0325 |
| Q5 | 0.1000 | 0.1796 | 0.0005 | 0.0091 | 0.1070 | 0.7394 | 0.0075 | 0.0443 |

Supplementary Table 2. Summary of estimation performance for the seroconversion rate (SCR) across sampling strategies grouped by relative precision (Q1–Q5) under a stable SCR scenario with a total sample size of 500. Q1 represents the top 20% of sampling structures with the highest precision, and Q5 the lowest. The grouping into Q1–Q5 was determined based on relative precision under the assumption of perfect assay performance. Performance metrics were then evaluated within each group under imperfect assay conditions assuming 90% sensitivity and specificity. Median estimates, relative precision, bias, and root mean squared error (RMSE) are calculated from the pooled distribution of estimates across simulations and sampling structures within each group, and are reported for both one-parameter and two-parameter models. Results are shown for four combinations of SCR and seroreversion rate (SRR): (A) SCR = 0.01 and SRR = 0.017, (B) SCR = 0.01 and SRR = 0.05, (C) SCR = 0.1 and SRR = 0.017, and (D) SCR = 0.1 and SRR = 0.05.

| **(A)** |  |  |  |  |  |  |  |  |
| --- | --- | --- | --- | --- | --- | --- | --- | --- |
|  | **1 parameter estimation** | | | | **2 parameters estimation** | | | |
|  | **Median** | **Relative Precision** | **Bias** | **RMSE** | **Median** | **Relative Precision** | **Bias** | **RMSE** |
| Q1 | 0.0130 | 0.2384 | 0.0031 | 0.0033 | 0.0329 | 1.4755 | 0.0219 | 0.0232 |
| Q2 | 0.0137 | 0.2569 | 0.0038 | 0.0040 | 0.0299 | 1.4424 | 0.0192 | 0.0207 |
| Q3 | 0.0146 | 0.2883 | 0.0046 | 0.0048 | 0.0294 | 1.5173 | 0.0187 | 0.0204 |
| Q4 | 0.0155 | 0.3279 | 0.0056 | 0.0058 | 0.0245 | 1.5578 | 0.0146 | 0.0171 |
| Q5 | 0.0183 | 0.7359 | 0.0090 | 0.0097 | 0.0217 | 1.6392 | 0.0123 | 0.0157 |
|  |  |  |  |  |  |  |  |  |
| **(B)** |  |  |  |  |  |  |  |  |
|  | **1 parameter estimation** | | | | **2 parameters estimation** | | | |
|  | **Median** | **Relative Precision** | **Bias** | **RMSE** | **Median** | **Relative Precision** | **Bias** | **RMSE** |
| Q1 | 0.0161 | 0.3425 | 0.0062 | 0.0064 | 0.0289 | 1.2879 | 0.0188 | 0.0199 |
| Q2 | 0.0169 | 0.3744 | 0.0070 | 0.0073 | 0.0259 | 1.2271 | 0.0154 | 0.0165 |
| Q3 | 0.0176 | 0.4003 | 0.0077 | 0.0080 | 0.0256 | 1.3108 | 0.0150 | 0.0164 |
| Q4 | 0.0188 | 0.4642 | 0.0090 | 0.0093 | 0.0234 | 1.3020 | 0.0126 | 0.0142 |
| Q5 | 0.0219 | 0.7961 | 0.0125 | 0.0132 | 0.0232 | 1.3876 | 0.0122 | 0.0142 |
|  |  |  |  |  |  |  |  |  |
| **(C)** |  |  |  |  |  |  |  |  |
|  | **1 parameter estimation** | | | | **2 parameters estimation** | | | |
|  | **Median** | **Relative Precision** | **Bias** | **RMSE** | **Median** | **Relative Precision** | **Bias** | **RMSE** |
| Q1 | 0.0883 | 0.1801 | -0.0107 | 0.0140 | 0.1153 | 0.3402 | 0.0169 | 0.0242 |
| Q2 | 0.0862 | 0.1830 | -0.0126 | 0.0157 | 0.1115 | 0.4085 | 0.0141 | 0.0255 |
| Q3 | 0.0842 | 0.1796 | -0.0147 | 0.0174 | 0.1092 | 0.4818 | 0.0134 | 0.0289 |
| Q4 | 0.0801 | 0.1668 | -0.0189 | 0.0207 | 0.1048 | 0.6373 | 0.0115 | 0.0357 |
| Q5 | 0.0736 | 0.1383 | -0.0260 | 0.0269 | 0.1004 | 1.1784 | 0.0313 | 0.0793 |
|  |  |  |  |  |  |  |  |  |
| **(D)** |  |  |  |  |  |  |  |  |
|  | **1 parameter estimation** | | | | **2 parameters estimation** | | | |
|  | **Median** | **Relative Precision** | **Bias** | **RMSE** | **Median** | **Relative Precision** | **Bias** | **RMSE** |
| Q1 | 0.0959 | 0.1722 | -0.0034 | 0.0094 | 0.1250 | 0.3446 | 0.0250 | 0.0308 |
| Q2 | 0.0964 | 0.1931 | -0.0027 | 0.0103 | 0.1211 | 0.4188 | 0.0217 | 0.0311 |
| Q3 | 0.0929 | 0.1758 | -0.0065 | 0.0111 | 0.1188 | 0.4864 | 0.0198 | 0.0329 |
| Q4 | 0.0930 | 0.1900 | -0.0061 | 0.0114 | 0.1035 | 0.5285 | 0.0077 | 0.0306 |
| Q5 | 0.0897 | 0.1708 | -0.0098 | 0.0131 | 0.1048 | 0.6594 | -0.0001 | 0.0382 |

Supplementary Table 3. Summary of estimation performance for the seroconversion rate (SCR) across sampling strategies grouped by relative precision (Q1–Q5) under a stable SCR scenario with a total sample size of 500. Q1 represents the top 20% of sampling structures with the highest precision, and Q5 the lowest. The grouping into Q1–Q5 was determined based on relative precision under the assumption of perfect assay performance. Performance metrics were then evaluated under imperfect assay conditions assuming 90% sensitivity and specificity, with SCR parameters estimated using a misclassification-adjusted likelihood that incorporates sensitivity and specificity to account for misclassification in the observed serostatus. Median estimates, relative precision, bias, and root mean squared error (RMSE) are calculated from the pooled distribution of estimates across simulations and sampling structures within each group, and are reported for both one-parameter and two-parameter models. Results are shown for four combinations of SCR and seroreversion rate (SRR): (A) SCR = 0.01 and SRR = 0.017, (B) SCR = 0.01 and SRR = 0.05, (C) SCR = 0.1 and SRR = 0.017, and (D) SCR = 0.1 and SRR = 0.05.

| **(A)** |  |  |  |  |  |  |  |  |
| --- | --- | --- | --- | --- | --- | --- | --- | --- |
|  | **1 parameter estimation** | | | | **2 parameters estimation** | | | |
|  | **Median** | **Relative Precision** | **Bias** | **RMSE** | **Median** | **Relative Precision** | **Bias** | **RMSE** |
| Q1 | 0.0100 | 0.2593 | 0.0000 | 0.0013 | 0.0103 | 0.8273 | 0.0012 | 0.0045 |
| Q2 | 0.0100 | 0.2771 | 0.0000 | 0.0014 | 0.0102 | 0.8633 | 0.0015 | 0.0049 |
| Q3 | 0.0100 | 0.3019 | 0.0000 | 0.0016 | 0.0102 | 0.9407 | 0.0018 | 0.0054 |
| Q4 | 0.0100 | 0.3316 | 0.0000 | 0.0017 | 0.0102 | 0.9940 | 0.0022 | 0.0060 |
| Q5 | 0.0100 | 0.4388 | 0.0000 | 0.0022 | 0.0101 | 1.1709 | 0.0032 | 0.0078 |
|  |  |  |  |  |  |  |  |  |
| **(B)** |  |  |  |  |  |  |  |  |
|  | **1 parameter estimation** | | | | **2 parameters estimation** | | | |
|  | **Median** | **Relative Precision** | **Bias** | **RMSE** | **Median** | **Relative Precision** | **Bias** | **RMSE** |
| Q1 | 0.0100 | 0.3588 | 0.0000 | 0.0018 | 0.0095 | 0.7624 | 0.0000 | 0.0041 |
| Q2 | 0.0100 | 0.3840 | 0.0000 | 0.0019 | 0.0097 | 0.7641 | 0.0001 | 0.0043 |
| Q3 | 0.0100 | 0.3958 | 0.0000 | 0.0020 | 0.0098 | 0.7849 | 0.0001 | 0.0044 |
| Q4 | 0.0100 | 0.4331 | 0.0001 | 0.0022 | 0.0099 | 0.7857 | 0.0001 | 0.0046 |
| Q5 | 0.0100 | 0.5360 | 0.0001 | 0.0027 | 0.0100 | 0.8163 | 0.0000 | 0.0049 |
|  |  |  |  |  |  |  |  |  |
| **(C)** |  |  |  |  |  |  |  |  |
|  | **1 parameter estimation** | | | | **2 parameters estimation** | | | |
|  | **Median** | **Relative Precision** | **Bias** | **RMSE** | **Median** | **Relative Precision** | **Bias** | **RMSE** |
| Q1 | 0.1001 | 0.1839 | 0.0006 | 0.0094 | 0.0999 | 0.2945 | 0.0012 | 0.0151 |
| Q2 | 0.0998 | 0.1986 | 0.0006 | 0.0102 | 0.0999 | 0.3611 | 0.0024 | 0.0196 |
| Q3 | 0.1000 | 0.2160 | 0.0008 | 0.0110 | 0.1002 | 0.4391 | 0.0041 | 0.0255 |
| Q4 | 0.1001 | 0.2353 | 0.0011 | 0.0120 | 0.0999 | 0.6626 | 0.0083 | 0.0442 |
| Q5 | 0.1001 | 0.2755 | 0.0018 | 0.0144 | 0.1036 | 1.7880 | 0.0641 | 0.1360 |
|  |  |  |  |  |  |  |  |  |
| **(D)** |  |  |  |  |  |  |  |  |
|  | **1 parameter estimation** | | | | **2 parameters estimation** | | | |
|  | **Median** | **Relative Precision** | **Bias** | **RMSE** | **Median** | **Relative Precision** | **Bias** | **RMSE** |
| Q1 | 0.1000 | 0.1933 | 0.0005 | 0.0099 | 0.1001 | 0.3723 | 0.0019 | 0.0190 |
| Q2 | 0.1000 | 0.2042 | 0.0006 | 0.0105 | 0.1002 | 0.4653 | 0.0034 | 0.0241 |
| Q3 | 0.1000 | 0.2141 | 0.0007 | 0.0109 | 0.1002 | 0.5679 | 0.0048 | 0.0300 |
| Q4 | 0.1001 | 0.2153 | 0.0007 | 0.0110 | 0.1000 | 0.6763 | 0.0090 | 0.0387 |
| Q5 | 0.0999 | 0.2272 | 0.0008 | 0.0116 | 0.1061 | 0.8224 | 0.0081 | 0.0498 |

Supplementary Table 4. Summary of estimation performance for the seroconversion rates (SCRs) before and after the change point, across sampling strategies grouped by joint precision (Q1–Q5) under a scenario assuming a single change point in transmission, with a total sample size of 1,000. The SCR was set to 0.03 before and 0.01 after the change point, and the seroreversion rate (SRR) was fixed at 0.017. Median estimates, relative precision, bias, and root mean squared error (RMSE) are calculated from the pooled distribution of estimates across simulations and sampling structures within each group. Results are shown for two model specifications: (A) two-parameter estimation, in which SCR before and after the change point are estimated, and (B) three-parameter estimation, in which SCR before, SCR after, and the seroreversion rate (SRR) are jointly estimated.

| **(A) 2 parametes estimation** | | |  |  |  |  |  |  |
| --- | --- | --- | --- | --- | --- | --- | --- | --- |
|  | **SCR before the change** | | | | **SCR after the change** | | | |
|  | **Median** | **Relative Precision** | **Bias** | **RMSE** | **Median** | **Relative Precision** | **Bias** | **RMSE** |
| τ = 3 years |  |  |  |  |  |  |  |  |
| Q1 | 0.0300 | 0.2093 | 0.0001 | 0.0032 | 0.0098 | 0.5874 | 0.0000 | 0.0030 |
| Q2 | 0.0300 | 0.1769 | 0.0000 | 0.0027 | 0.0099 | 0.6790 | 0.0001 | 0.0035 |
| Q3 | 0.0300 | 0.2145 | 0.0000 | 0.0033 | 0.0098 | 0.7893 | 0.0000 | 0.0040 |
| Q4 | 0.0300 | 0.1593 | 0.0001 | 0.0024 | 0.0098 | 0.9003 | 0.0000 | 0.0048 |
| Q5 | 0.0300 | 0.1649 | 0.0000 | 0.0025 | 0.0099 | 0.9336 | 0.0001 | 0.0049 |
| τ = 5 years |  |  |  |  |  |  |  |  |
| Q1 | 0.0300 | 0.2222 | 0.0001 | 0.0034 | 0.0099 | 0.4661 | 0.0000 | 0.0024 |
| Q2 | 0.0300 | 0.1780 | 0.0001 | 0.0027 | 0.0099 | 0.5733 | 0.0000 | 0.0029 |
| Q3 | 0.0300 | 0.2254 | 0.0000 | 0.0034 | 0.0099 | 0.6331 | 0.0000 | 0.0032 |
| Q4 | 0.0300 | 0.2227 | 0.0000 | 0.0034 | 0.0099 | 0.7142 | 0.0000 | 0.0036 |
| Q5 | 0.0301 | 0.1770 | 0.0000 | 0.0027 | 0.0098 | 0.7776 | 0.0000 | 0.0041 |
| τ = 10 years |  |  |  |  |  |  |  |  |
| Q1 | 0.0301 | 0.2398 | 0.0002 | 0.0036 | 0.0099 | 0.3686 | 0.0000 | 0.0019 |
| Q2 | 0.0299 | 0.2465 | 0.0000 | 0.0037 | 0.0100 | 0.4086 | 0.0000 | 0.0021 |
| Q3 | 0.0300 | 0.2378 | 0.0001 | 0.0036 | 0.0099 | 0.4741 | 0.0000 | 0.0024 |
| Q4 | 0.0300 | 0.2562 | 0.0001 | 0.0038 | 0.0099 | 0.5295 | 0.0000 | 0.0027 |
| Q5 | 0.0300 | 0.3084 | 0.0000 | 0.0046 | 0.0099 | 0.5847 | 0.0001 | 0.0030 |
|  |  |  |  |  |  |  |  |  |
| **(B) 3 parametes estimation** | | |  |  |  |  |  |  |
|  | **SCR before the change** | | | | **SCR after the change** | | | |
|  | **Median** | **Relative Precision** | **Bias** | **RMSE** | **Median** | **Relative Precision** | **Bias** | **RMSE** |
| τ = 3 years |  |  |  |  |  |  |  |  |
| Q1 | 0.0300 | 0.5208 | 0.0000 | 0.0028 | 0.0098 | 0.6951 | 0.0000 | 0.0035 |
| Q2 | 0.0300 | 0.5864 | 0.0001 | 0.0030 | 0.0097 | 0.8272 | 0.0000 | 0.0042 |
| Q3 | 0.0300 | 0.6161 | 0.0001 | 0.0030 | 0.0099 | 0.8654 | 0.0001 | 0.0045 |
| Q4 | 0.0300 | 0.9292 | 0.0000 | 0.0028 | 0.0098 | 0.8445 | 0.0000 | 0.0044 |
| Q5 | 0.0300 | 2.2824 | 0.0001 | 0.0025 | 0.0101 | 0.8113 | 0.0000 | 0.0040 |
| τ = 5 years |  |  |  |  |  |  |  |  |
| Q1 | 0.0300 | 0.5923 | 0.0001 | 0.0031 | 0.0099 | 0.5822 | 0.0000 | 0.0029 |
| Q2 | 0.0300 | 0.5911 | 0.0000 | 0.0027 | 0.0099 | 0.7093 | 0.0000 | 0.0036 |
| Q3 | 0.0300 | 0.8424 | 0.0000 | 0.0032 | 0.0098 | 0.7084 | 0.0000 | 0.0036 |
| Q4 | 0.0300 | 1.1994 | 0.0000 | 0.0037 | 0.0099 | 0.6867 | 0.0000 | 0.0034 |
| Q5 | 0.0300 | 4.1598 | 0.0001 | 0.0029 | 0.0098 | 0.6473 | 0.0000 | 0.0031 |
| τ = 10 years |  |  |  |  |  |  |  |  |
| Q1 | 0.0300 | 0.7865 | 0.0001 | 0.0036 | 0.0099 | 0.4746 | 0.0000 | 0.0024 |
| Q2 | 0.0301 | 0.9150 | 0.0001 | 0.0034 | 0.0099 | 0.5135 | 0.0000 | 0.0026 |
| Q3 | 0.0300 | 1.3134 | 0.0001 | 0.0037 | 0.0099 | 0.5159 | 0.0000 | 0.0026 |
| Q4 | 0.0299 | 3.3000 | 0.0000 | 0.0051 | 0.0100 | 0.4830 | 0.0001 | 0.0021 |
| Q5 | 0.0300 | 10.9702 | 0.0001 | 0.0033 | 0.0099 | 0.5361 | 0.0000 | 0.0025 |

Supplementary Table 5. Summary of estimation performance for the seroconversion rates (SCRs) before and after the change point, across sampling strategies grouped by joint precision (Q1–Q5) under a scenario assuming a single change point in transmission, with a total sample size of 1,000. The SCR was set to 0.03 before and 0.01 after the change point, and the seroreversion rate (SRR) was fixed at 0.017. The grouping into Q1–Q5 was determined based on relative joint precision under the assumption of perfect assay performance. Performance metrics were then evaluated within each group under imperfect assay conditions assuming 90% sensitivity and specificity. Median estimates, relative precision, bias, and root mean squared error (RMSE) are calculated from the pooled distribution of estimates across simulations and sampling structures within each group. Results are shown for two model specifications: (A) two-parameter estimation, in which SCR before and after the change point are estimated, and (B) three-parameter estimation, in which SCR before, SCR after, and the seroreversion rate (SRR) are jointly estimated.

| **(A) 2 parametes estimation** | | |  |  |  |  |  |  |
| --- | --- | --- | --- | --- | --- | --- | --- | --- |
|  | **SCR before the change** | | | | **SCR after the change** | | | |
|  | **Median** | **Relative Precision** | **Bias** | **RMSE** | **Median** | **Relative Precision** | **Bias** | **RMSE** |
| τ = 3 years |  |  |  |  |  |  |  |  |
| Q1 | 0.0222 | 0.2260 | -0.0077 | 0.0084 | 0.0619 | 1.5067 | 0.0521 | 0.0527 |
| Q2 | 0.0222 | 0.2228 | -0.0079 | 0.0086 | 0.0609 | 1.7186 | 0.0513 | 0.0520 |
| Q3 | 0.0224 | 0.2999 | -0.0080 | 0.0091 | 0.0579 | 1.7556 | 0.0480 | 0.0488 |
| Q4 | 0.0227 | 0.2006 | -0.0073 | 0.0079 | 0.0585 | 2.3279 | 0.0495 | 0.0508 |
| Q5 | 0.0225 | 0.2282 | -0.0076 | 0.0083 | 0.0589 | 2.3145 | 0.0496 | 0.0510 |
| τ = 5 years |  |  |  |  |  |  |  |  |
| Q1 | 0.0211 | 0.2443 | -0.0089 | 0.0096 | 0.0445 | 1.0518 | 0.0346 | 0.0350 |
| Q2 | 0.0213 | 0.2053 | -0.0087 | 0.0092 | 0.0435 | 1.2308 | 0.0338 | 0.0344 |
| Q3 | 0.0216 | 0.3217 | -0.0089 | 0.0100 | 0.0403 | 1.2032 | 0.0304 | 0.0310 |
| Q4 | 0.0218 | 0.3009 | -0.0084 | 0.0095 | 0.0406 | 1.4492 | 0.0310 | 0.0318 |
| Q5 | 0.0212 | 0.2469 | -0.0089 | 0.0096 | 0.0436 | 1.7118 | 0.0341 | 0.0352 |
| τ = 10 years |  |  |  |  |  |  |  |  |
| Q1 | 0.0200 | 0.2558 | -0.0100 | 0.0107 | 0.0286 | 0.6802 | 0.0187 | 0.0190 |
| Q2 | 0.0196 | 0.2743 | -0.0104 | 0.0112 | 0.0285 | 0.7159 | 0.0186 | 0.0190 |
| Q3 | 0.0213 | 0.2773 | -0.0088 | 0.0097 | 0.0256 | 0.7531 | 0.0158 | 0.0162 |
| Q4 | 0.0207 | 0.3219 | -0.0095 | 0.0106 | 0.0262 | 0.9302 | 0.0166 | 0.0173 |
| Q5 | 0.0194 | 0.4265 | -0.0113 | 0.0128 | 0.0283 | 0.9923 | 0.0185 | 0.0192 |
|  |  |  |  |  |  |  |  |  |
| **(B) 3 parametes estimation** | | |  |  |  |  |  |  |
|  | **SCR before the change** | | | | **SCR after the change** | | | |
|  | **Median** | **Relative Precision** | **Bias** | **RMSE** | **Median** | **Relative Precision** | **Bias** | **RMSE** |
| τ = 3 years |  |  |  |  |  |  |  |  |
| Q1 | 0.0224 | 0.6181 | -0.0075 | 0.0082 | 0.0593 | 1.6441 | 0.0495 | 0.0502 |
| Q2 | 0.0224 | 0.5898 | -0.0079 | 0.0089 | 0.0580 | 1.9852 | 0.0483 | 0.0492 |
| Q3 | 0.0226 | 0.6788 | -0.0076 | 0.0086 | 0.0586 | 2.0667 | 0.0488 | 0.0499 |
| Q4 | 0.0222 | 0.9265 | -0.0079 | 0.0087 | 0.0608 | 2.2806 | 0.0511 | 0.0522 |
| Q5 | 0.0225 | 2.1965 | -0.0075 | 0.0080 | 0.0625 | 2.2708 | 0.0529 | 0.0539 |
| τ = 5 years |  |  |  |  |  |  |  |  |
| Q1 | 0.0215 | 0.7509 | -0.0084 | 0.0091 | 0.0416 | 1.2377 | 0.0316 | 0.0322 |
| Q2 | 0.0220 | 0.6747 | -0.0080 | 0.0086 | 0.0409 | 1.4176 | 0.0313 | 0.0321 |
| Q3 | 0.0213 | 0.9315 | -0.0090 | 0.0100 | 0.0420 | 1.6069 | 0.0324 | 0.0332 |
| Q4 | 0.0205 | 1.4167 | -0.0099 | 0.0111 | 0.0432 | 1.7051 | 0.0335 | 0.0342 |
| Q5 | 0.0214 | 4.0918 | -0.0086 | 0.0092 | 0.0451 | 1.7205 | 0.0353 | 0.0359 |
| τ = 10 years |  |  |  |  |  |  |  |  |
| Q1 | 0.0210 | 1.1202 | -0.0089 | 0.0097 | 0.0262 | 0.9418 | 0.0164 | 0.0168 |
| Q2 | 0.0208 | 4.9404 | -0.0092 | 0.0100 | 0.0269 | 1.1442 | 0.0171 | 0.0177 |
| Q3 | 0.0204 | 2.3940 | -0.0097 | 0.0106 | 0.0269 | 1.2707 | 0.0171 | 0.0177 |
| Q4 | 0.0182 | 6.7484 | -0.0124 | 0.0139 | 0.0282 | 1.2830 | 0.0183 | 0.0187 |
| Q5 | 0.0201 | 13.3682 | -0.0098 | 0.0105 | 0.0293 | 1.4212 | 0.0195 | 0.0200 |

Supplementary Table 6. Summary of estimation performance for the seroconversion rates (SCRs) before and after the change point, across sampling strategies grouped by joint precision (Q1–Q5) under a scenario assuming a single change point in transmission, with a total sample size of 1,000. The SCR was set to 0.03 before and 0.01 after the change point, and the seroreversion rate (SRR) was fixed at 0.017. The grouping into Q1–Q5 was determined based on relative precision under the assumption of perfect assay performance. Performance metrics were then evaluated under imperfect assay conditions assuming 90% sensitivity and specificity, with SCR parameters estimated using a misclassification-adjusted likelihood that incorporates sensitivity and specificity to account for misclassification in the observed serostatus. Median estimates, relative precision, bias, and root mean squared error (RMSE) are calculated from the pooled distribution of estimates across simulations and sampling structures within each group. Results are shown for two model specifications: (A) two-parameter estimation, in which SCR before and after the change point are estimated, and (B) three-parameter estimation, in which SCR before, SCR after, and the seroreversion rate (SRR) are jointly estimated.

| **(A) 2 parametes estimation** | | |  |  |  |  |  |  |
| --- | --- | --- | --- | --- | --- | --- | --- | --- |
|  | **SCR before the change** | | | | **SCR after the change** | | | |
|  | **Median** | **Relative Precision** | **Bias** | **RMSE** | **Median** | **Relative Precision** | **Bias** | **RMSE** |
| τ = 3 years |  |  |  |  |  |  |  |  |
| Q1 | 0.0300 | 0.2795 | 0.0001 | 0.0042 | 0.0098 | 1.3131 | 0.0004 | 0.0074 |
| Q2 | 0.0299 | 0.2533 | 0.0000 | 0.0039 | 0.0098 | 1.4476 | 0.0006 | 0.0083 |
| Q3 | 0.0299 | 0.3220 | -0.0001 | 0.0048 | 0.0099 | 1.5088 | 0.0008 | 0.0086 |
| Q4 | 0.0298 | 0.2289 | -0.0002 | 0.0035 | 0.0098 | 1.8008 | 0.0015 | 0.0106 |
| Q5 | 0.0298 | 0.2507 | -0.0003 | 0.0038 | 0.0099 | 1.8047 | 0.0016 | 0.0107 |
| τ = 5 years |  |  |  |  |  |  |  |  |
| Q1 | 0.0300 | 0.3017 | 0.0002 | 0.0045 | 0.0098 | 1.0757 | 0.0000 | 0.0054 |
| Q2 | 0.0300 | 0.2533 | 0.0001 | 0.0039 | 0.0098 | 1.1633 | 0.0001 | 0.0062 |
| Q3 | 0.0299 | 0.3326 | 0.0000 | 0.0050 | 0.0099 | 1.1674 | 0.0002 | 0.0063 |
| Q4 | 0.0299 | 0.3171 | 0.0000 | 0.0049 | 0.0100 | 1.2878 | 0.0004 | 0.0070 |
| Q5 | 0.0299 | 0.2790 | -0.0002 | 0.0043 | 0.0098 | 1.4767 | 0.0008 | 0.0084 |
| τ = 10 years |  |  |  |  |  |  |  |  |
| Q1 | 0.0301 | 0.3240 | 0.0002 | 0.0049 | 0.0099 | 0.6888 | 0.0000 | 0.0035 |
| Q2 | 0.0300 | 0.3352 | 0.0000 | 0.0051 | 0.0100 | 0.7353 | 0.0001 | 0.0037 |
| Q3 | 0.0301 | 0.3400 | 0.0002 | 0.0052 | 0.0100 | 0.8095 | 0.0000 | 0.0041 |
| Q4 | 0.0300 | 0.3718 | 0.0001 | 0.0056 | 0.0099 | 0.9491 | 0.0001 | 0.0047 |
| Q5 | 0.0301 | 0.4350 | 0.0000 | 0.0066 | 0.0099 | 1.0559 | 0.0001 | 0.0052 |
|  |  |  |  |  |  |  |  |  |
| **(B) 3 parametes estimation** | | |  |  |  |  |  |  |
|  | **SCR before the change** | | | | **SCR after the change** | | | |
|  | **Median** | **Relative Precision** | **Bias** | **RMSE** | **Median** | **Relative Precision** | **Bias** | **RMSE** |
| τ = 3 years |  |  |  |  |  |  |  |  |
| Q1 | 0.0299 | 0.7474 | 0.0000 | 0.0039 | 0.0099 | 1.3875 | 0.0006 | 0.0080 |
| Q2 | 0.0299 | 0.7798 | -0.0001 | 0.0044 | 0.0098 | 1.5735 | 0.0009 | 0.0090 |
| Q3 | 0.0299 | 0.8646 | -0.0001 | 0.0044 | 0.0098 | 1.6260 | 0.0011 | 0.0095 |
| Q4 | 0.0299 | 1.1576 | -0.0002 | 0.0041 | 0.0099 | 1.6876 | 0.0013 | 0.0099 |
| Q5 | 0.0299 | 2.4963 | -0.0001 | 0.0034 | 0.0097 | 1.6906 | 0.0010 | 0.0096 |
| τ = 5 years |  |  |  |  |  |  |  |  |
| Q1 | 0.0299 | 0.8487 | 0.0001 | 0.0044 | 0.0098 | 1.1240 | 0.0001 | 0.0059 |
| Q2 | 0.0300 | 0.8490 | 0.0000 | 0.0039 | 0.0099 | 1.2719 | 0.0004 | 0.0070 |
| Q3 | 0.0299 | 1.1738 | 0.0000 | 0.0047 | 0.0098 | 1.3114 | 0.0004 | 0.0072 |
| Q4 | 0.0299 | 1.7121 | -0.0001 | 0.0055 | 0.0099 | 1.2651 | 0.0005 | 0.0069 |
| Q5 | 0.0300 | 4.3282 | 0.0001 | 0.0040 | 0.0098 | 1.2934 | 0.0003 | 0.0067 |
| τ = 10 years |  |  |  |  |  |  |  |  |
| Q1 | 0.0300 | 1.1484 | 0.0002 | 0.0051 | 0.0100 | 0.8400 | 0.0000 | 0.0041 |
| Q2 | 0.0300 | 1.3542 | 0.0001 | 0.0049 | 0.0100 | 0.9249 | 0.0001 | 0.0045 |
| Q3 | 0.0301 | 2.1349 | 0.0001 | 0.0054 | 0.0099 | 0.9403 | 0.0001 | 0.0045 |
| Q4 | 0.0299 | 5.4155 | 0.0000 | 0.0073 | 0.0100 | 0.8777 | 0.0001 | 0.0039 |
| Q5 | 0.0300 | 12.6210 | 0.0002 | 0.0046 | 0.0099 | 0.9977 | 0.0001 | 0.0045 |

# Supplementary Figures

Supplementary Figure 1**.** The 126 possible combinations of five age groups ([1,10], [11, 20], [21, 30], [31, 40], and [41, 50]) with proportions ranging from 0% to 100% in increments of 20%.

Supplementary Figure 2**.** The 69 possible combinations of five age groups ([1, $\tau$], [$\tau+1$, $\tau+10$], [$\tau+11$, $\tau+20$], [$\tau+21$, $\tau+30$], and [$\tau+31$, $\tau+40$]) under a single change point in malaria transmission intensity scenario. $\tau$ corresponds the time (in years) since the change point. For a given value of τ, these correspond to fixed age intervals (e.g., [1, 10], [11, 20], [21, 30], [31, 40], and [41, 50] when τ = 10). The proportion of the first age group was varied from 20 to 80% in increments of 20%, while the other age groups were varied from 0 to 80% to capture information from both before and after the change point.

Supplementary Figure 3**.** Age-based sampling structures that yielded the bottom 20% relative precision under the stable malaria transmission intensity scenario with a total sample size of 500. Panels show results for (A) estimation of the seroconversion rate (SCR) only, and (B) joint estimation of SCR and the seroreversion rate (SRR). Each row represents a distinct sampling strategy, ordered by relative precision, with lower values indicating higher precision (i.e., highest precision at the top). Columns indicate age groups, and colours represent the proportion of the total sample allocated to each age group. The additional column on the right shows the relative precision associated with each sampling strategy.

Supplementary Figure 4**.** Age-based sampling structures that yielded the top 20% relative precision under the stable malaria transmission intensity scenario with a total sample size of 1,000. Panels show results for (A) estimation of the seroconversion rate (SCR) only, and (B) joint estimation of SCR and the seroreversion rate (SRR). Each row represents a distinct sampling strategy, ordered by relative precision, with lower values indicating higher precision (i.e., highest precision at the top). Columns indicate age groups, and colours represent the proportion of the total sample allocated to each age group. The additional column on the right shows the relative precision associated with each sampling strategy.

Supplementary Figure 5**.** Age-based sampling structures that yielded the bottom 20% relative precision under the stable malaria transmission intensity scenario with a total sample size of 1,000. Panels show results for (A) estimation of the seroconversion rate (SCR) only, and (B) joint estimation of SCR and the seroreversion rate (SRR). Each row represents a distinct sampling strategy, ordered by relative precision, with lower values indicating higher precision (i.e., highest precision at the top). Columns indicate age groups, and colours represent the proportion of the total sample allocated to each age group. The additional column on the right shows the relative precision associated with each sampling strategy.

Supplementary Figure 6**.** Relative precisions (A and B) and empirical 95% intervals (C and D) of the estimated seroconversion rates (SCRs) before (A and C) and after (B and D) a single change point in malaria transmission intensity. The analysis assumed a total sample size of 1,000 with low underlying SCRs (SCR = 0.1 before and 0.03 after the change point) and a low seroreversion rate (SRR = 0.017). Results are stratified by 1) the time since the change point ($\tau$ = 3, 5, and 10 years, representing the time between the change point and data collection) and 2) the number of parameters estimated (2 parameter: SCRs before and after the change point; 3 parameters: SCRs and SRR). Panels C and D are further stratified by the age structures that yielded the top 20% joint precision (Q1) and the bottom 20% joint precision (Q5). Each dot in Panels C and D represents the median estimated SCR, and error bars indicate the empirical 95% interval based on the pooled distribution of estimates across simulations and sampling structures within each group. Note that the y-axis scales differ across panels to reflect differences in parameter magnitude and model specification.

Supplementary Figure 7**.** Age-based sampling structures that yielded the bottom 20% joint precision under a single change point in malaria transmission intensity scenario. The analysis assumed a total sample size of 1,000 with low underlying SCRs (SCR = 0.03 before and 0.01 after the change point) and a low seroreversion rate (SRR = 0.017). Results were stratified by the time since the change point (3, 5, and 10 years). A) Only the SCR before and after the change point were estimated. B) The SCRs and the seroreversion rate (SRR) were jointly estimated. Each row represents a distinct sampling strategy, ordered by relative precision, with lower values indicating higher precision (i.e., highest precision at the top). Columns indicate age groups, and colours represent the proportion of the total sample allocated to each age group. The additional column on the right shows the relative precision associated with each sampling strategy.

Supplementary Figure 8**.** Age-based sampling structures that yielded the top 20% joint precision under a single change point in malaria transmission intensity scenario. The analysis assumed a total sample size of 1,000 with high underlying SCRs (SCR = 0.1 before and 0.03 after the change point) and a low seroreversion rate (SRR = 0.017). Results were stratified by the time since the change point (3, 5, and 10 years). A) Only the SCR before and after the change point were estimated. B) The SCRs and the seroreversion rate (SRR) were jointly estimated. Each row represents a distinct sampling strategy, ordered by relative precision, with lower values indicating higher precision (i.e., highest precision at the top). Columns indicate age groups, and colours represent the proportion of the total sample allocated to each age group. The additional column on the right shows the relative precision associated with each sampling strategy.

Supplementary Figure 9**.** Age-based sampling structures that yielded the bottom 20% joint precision under a single change point in malaria transmission intensity scenario. The analysis assumed a total sample size of 1,000 with high underlying SCRs (SCR = 0.1 before and 0.03 after the change point) and a low seroreversion rate (SRR = 0.017). Results were stratified by the time since the change point (3, 5, and 10 years). A) Only the SCR before and after the change point were estimated. B) The SCRs and the seroreversion rate (SRR) were jointly estimated. Each row represents a distinct sampling strategy, ordered by relative precision, with lower values indicating higher precision (i.e., highest precision at the top). Columns indicate age groups, and colours represent the proportion of the total sample allocated to each age group. The additional column on the right shows the relative precision associated with each sampling strategy.

Supplementary Figure 10**.** Statistical power to detect a change in seroconversion rate in high transmission settings (SCR, 0.1 before and 0.03 after the change point). Solid lines represent the fitted logistic functions applied to the corresponding simulated results (filled circles), stratified by the quintiles of joint precision (Q1–Q5). A) Only the SCR before and after the change point were estimated. B) The SCRs and the seroreversion rate (SRR) were jointly estimated.

Supplementary Figure 11**.** The user interface (A) and output (B) of the Shiny application. By specifying the scenario, values for seroconversion rate (SCR) and seroreversion rate (SRR), the change point (for the chage-in-SCR scenario), and the number of parameters to be estimated, the user can obtain the 95% CI for the SCR(s) along with the age-based sampling structures that yield the highest and lowest precision.
